# Joint assessment of insulin resistance surrogate indices and basal metabolic rate for primary prevention of cardiometabolic multimorbidity: evidence from the China Health and Retirement Longitudinal Study (2011–2020)

**DOI:** 10.1186/s12933-025-03067-y

**Published:** 2026-01-12

**Authors:** Hurong Lai, Chao Deng, Caifeng Liao, Ruixue Tian, Kexin Liu, Zhangjun Luo, Ling He, Huaijun Tu, Jian Li

**Affiliations:** 1 The Second Affiliated Hospital, Jiangxi Medical College, Nanchang University, Nanchang, Jiangxi China; 2 Department of Clinical Trial Research Center, The Second Affiliated Hospital of Nanchang University, Nanchang, Jiangxi China; 3 Department of Geriatrics, The Second Affiliated Hospital of Nanchang University, Nanchang, Jiangxi China

**Keywords:** Insulin resistance, Basal metabolic rate, Cardiometabolic multimorbidity, CHARLS

## Abstract

**Background:**

The triglyceride-glucose (TyG) index, estimated glucose disposal rate (eGDR), and metabolic score for insulin resistance (METS-IR) are well-established surrogate indices of insulin resistance (IR). Although both IR and elevated basal metabolic rate (BMR) are recognized risk factors for cardiometabolic diseases, their joint effects on the risk of cardiometabolic multimorbidity (CMM) remain unclear. This study aimed to investigate the separate and combined associations of these IR surrogates and BMR with incident CMM.

**Methods:**

We included 7204 eligible participants from the 2011–2020 survey waves of the China Health and Retirement Longitudinal Study (CHARLS). Participants were stratified by median values of IR surrogate indices and BMR. The associations with CMM were assessed using Kaplan–Meier curves, multivariable Cox regression, and restricted cubic splines (RCS). Predictive performance was evaluated using receiver operating characteristic (ROC) curves, net reclassification improvement (NRI), and integrated discrimination improvement (IDI). Mediation and interaction analyses were performed to explore potential underlying relationships.

**Results:**

Over a median follow-up of 9 years, 1103 participants (15.31%) developed CMM. Compared to those with low IR and low BMR, participants with high levels of both exhibited the highest risk of CMM, with hazard ratios of 2.13 (95% CI 1.73–2.63) for TyG, 1.93 (95% CI 1.57–2.36) for eGDR, and 1.92 (95% CI 1.61–2.29) for METS-IR. These associations remained consistent in subgroup and sensitivity analyses. Adding IR indices and BMR to the baseline model significantly improved CMM prediction: TyG (AUC 0.759, NRI 0.371, IDI 0.017; all *P* < 0.001), eGDR (AUC 0.753, NRI 0.330, IDI 0.012; all *P* < 0.01), and METS-IR (AUC 0.747, NRI 0.170, IDI 0.004; all *P* < 0.01). Mediation analysis demonstrated that all IR indices significantly mediated the association between BMR and CMM, and a bidirectional mediation relationship was specifically observed between BMR and the TyG index. Notably, no significant additive or multiplicative interactions were detected.

**Conclusion:**

IR surrogate indices and BMR independently and jointly predicted the risk of CMM, with IR pathways substantially mediating the effect of BMR. The combined assessment of these parameters may improve CMM risk stratification and guide primary prevention strategies.

**Graphical abstract:**

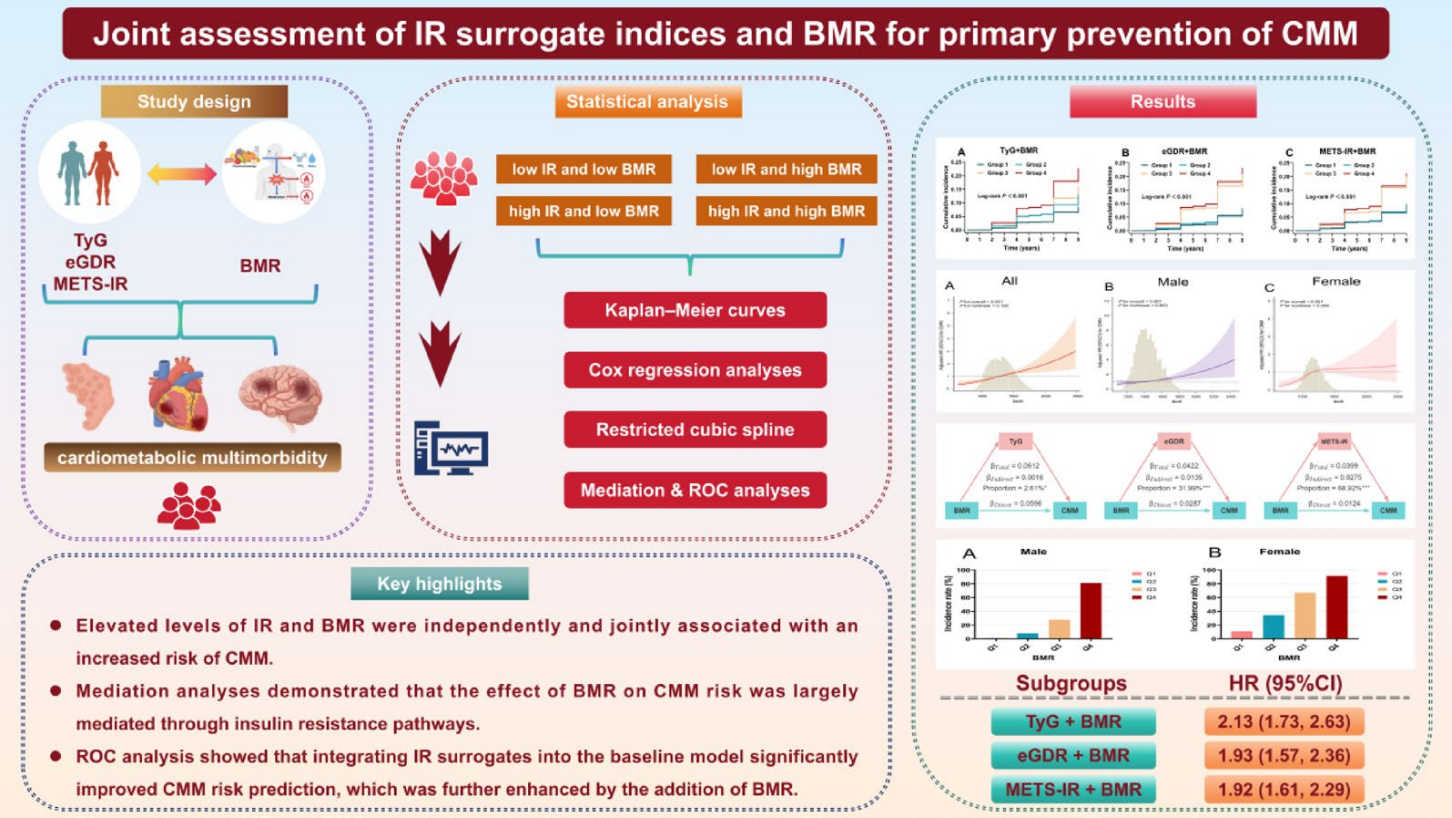

**Supplementary Information:**

The online version contains supplementary material available at 10.1186/s12933-025-03067-y.

## Research insights

**What is currently known about this topic?**

- The growing burden of population aging has contributed to a significant surge in the prevalence of CMM.
- CMM significantly diminishes quality of life, accelerates functional decline, and markedly increases mortality risk.
- Although both IR and elevated BMR are recognized risk factors for CMDs, their joint effects on the risk of CMM remain unclear.

**What is the key research question?**

Whether the coexistence of high levels of IR and BMR confers a more than additive risk, and whether their combination could improve early risk stratification for CMM.

**What is new?**

- Elevated IR and BMR levels were independently and jointly associated with an increased risk of CMM.
- Incorporating IR surrogate indices into the baseline model significantly improved CMM risk prediction, and the addition of BMR provided further incremental predictive value.
- Mediation analysis revealed that all IR surrogate indices significantly mediated the BMR-CMM relationship, whereas the mediating effect of BMR was observed only for the TyG index.

**How might this study influence clinical practice?**

Future preventive strategies, whether lifestyle-based or pharmacological, must target the stabilization of energy metabolism while enhancing insulin sensitivity to more effectively prevent CMM.

## Background

Multimorbidity, defined as the coexistence of two or more chronic conditions in an individual, represents a major global health challenge, particularly among adults aged 65 years and older [1]. Cardiometabolic multimorbidity (CMM), one of the most prevalent and severe forms of multimorbidity, is characterized by the concurrence of at least two cardiometabolic diseases (CMDs), such as diabetes, heart disease, and stroke [2]. A growing body of evidence indicates that CMM significantly diminishes quality of life, accelerates functional decline, and markedly increases mortality risk [3, 4]. Given its rising prevalence, there is an urgent need to identify modifiable risk factors and elucidate underlying pathogenic mechanisms to inform effective primary prevention strategies [5].

Insulin resistance (IR) is a well-established pathophysiological cornerstone of various CMDs [6], with key mechanisms involving endothelial dysfunction [7], oxidative stress [8], lipid metabolism dysregulation [9], and inflammatory pathway activation [10]. Although the hyperinsulinemic-euglycemic clamp remains the gold standard for assessing IR, its clinical utility is limited by complexity and cost. Consequently, several easily accessible surrogate indices, including the triglyceride-glucose (TyG) index [11], estimated glucose disposal rate (eGDR) [12], and metabolic score for insulin resistance (METS-IR) [13], have been developed and validated in large-scale epidemiological studies as reliable predictors of CMD incidence and mortality [14]. Concurrently, basal metabolic rate (BMR), which reflects the body’s energy expenditure at rest, has also been implicated in cardiometabolic health. Evidence from multiple large-scale cohort studies demonstrates that elevated BMR is significantly associated with an increased incidence of ischemic stroke, type 2 diabetes, and heart disease [15–17].

It is noteworthy that a close interaction may exist between BMR and IR. In humans, BMR accounts for 60–70% of total daily energy expenditure and plays a crucial role in energy balance [18]. An elevated BMR signifies increased energy demand, primarily met by mitochondrial activity. This heightened demand stimulates glycolytic flux, promoting the generation of reactive oxygen species (ROS) [19]. Excessive ROS, in turn, can induce endothelial dysfunction [20], chronic inflammation [21], and mitochondrial impairment [22], ultimately contributing to the development of IR [23]. Supporting this connection, one study reported a significant positive correlation between IR and BMR, suggesting a potential synergistic effect on health outcomes [24]. However, the joint effects of BMR and IR on the risk of CMM remains largely unexplored. It remains unclear whether the coexistence of high IR and high BMR confers a synergistic risk exceeding the sum of their individual effects, and whether their combination could enhance early risk stratification for CMM.

To address these critical knowledge gaps, we conducted a prospective cohort study using data from the China Health and Retirement Longitudinal Study (CHARLS). This study aims to: (1) investigate the separate and combined associations of three key IR surrogate indices (TyG, eGDR, and METS-IR) and BMR with the incident risk of CMM; (2) evaluate the incremental predictive value of integrating these IR indices with BMR for CMM risk; and (3) explore potential mediating and interaction effects between IR and BMR. Our findings are expected to provide novel epidemiological insights that could inform the development of more precise and effective preventive interventions for CMM.

## Methods

### Study design and participants

This community-based longitudinal analysis utilized data from the 2011 (baseline) and 2020 (follow-up) waves of the CHARLS, a publicly accessible dataset (http://charls.pku.edu.cn/). CHARLS is a prospective, nationally representative cohort study investigating health and economic transitions among adults aged 45 years and older in China. The baseline survey was conducted between June 2011 and March 2012, employing a multistage probability sampling strategy to recruit 17,708 participants from 450 communities across 28 provinces in China [25]. The high baseline response rate of 80.5% enhances the statistical power and minimizes potential selection bias, ensuring robust population representativeness. All anthropometric measurements and fasting venous blood samples were collected by trained medical staff following a standardized protocol. The CHARLS study was approved by the Peking University Biomedical Ethics Committee (IRB00001052-11015), and written informed consent was obtained from all participants prior to data collection.

**Fig. 1:**
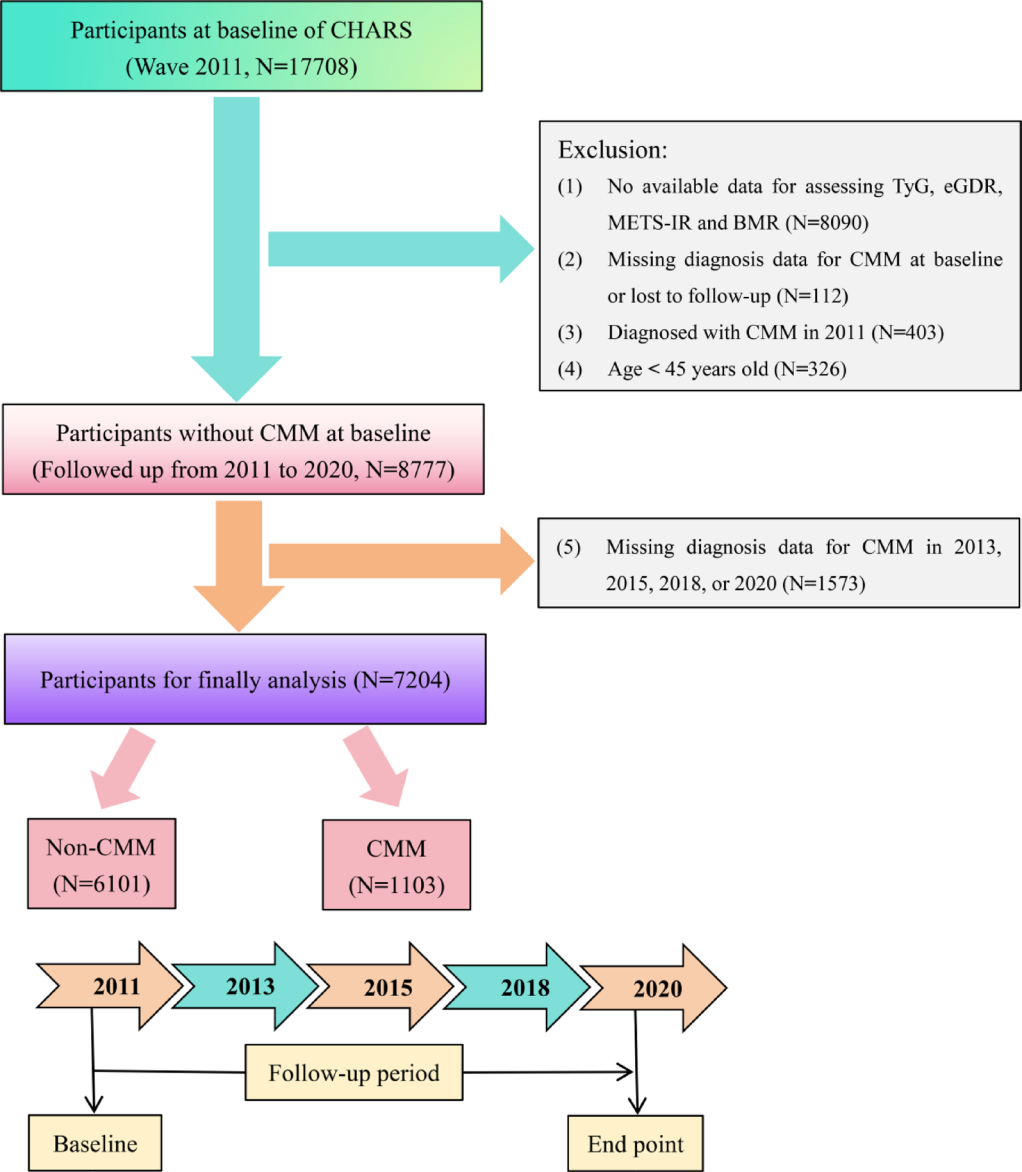
Flow chart of inclusion and exclusion criteria of participants

In the present investigation, the baseline survey initially included 17,708 individuals. The primary objective was to evaluate the potential joint or reciprocal mediating effects of BMR and IR on the development of CMM. To maintain analytical rigor, the study was restricted to individuals aged 45 years or older. A systematic exclusion process was applied as follows: (1) no available data for assessing TyG, eGDR, METS-IR and BMR (n = 8090); (2) missing diagnosis data for CMM at baseline or lost to follow-up (n = 112); (3) diagnosed with CMM in 2011 (N** = **403); (4) age < 45 years old (n = 326); (5) missing diagnosis data for CMM in 2013, 2015, 2018, or 2020 (n = 1573). After applying these criteria, 7204 participants were included in the final analysis. A flowchart detailing the participant selection process is provided in Fig. 1.

### Data collection and measurements

All anthropometric and laboratory assessments were conducted by trained staff using standardized protocols. A comprehensive dataset was assembled, encompassing key demographic characteristics, lifestyle factors, and health status. The collected variables included age, gender, height, body weight, waist circumference (WC), marital status, residential location (urban/rural), educational attainment, smoking status, alcohol consumption, and history of physician-diagnosed conditions—specifically hypertension, diabetes, heart disease, and stroke. Body weight was measured without footwear to the nearest 0.1 kg. Height was determined using a stadiometer and recorded to the nearest 0.1 cm. WC was measured horizontally at the umbilical level with a precision of 0.1 cm. Resting blood pressure was calculated as the mean of three consecutive readings obtained with an Omron HEM-7200 monitor.

After an overnight fast, venous blood samples were collected by qualified medical staff. All samples were aliquoted and stored at − 20 °C before being transported under monitored cold-chain conditions to the central laboratory at the Chinese Center for Disease Control and Prevention in Beijing for standardized analysis. Serum concentrations of triglycerides (TG), total cholesterol (TC), low-density lipoprotein cholesterol (LDL-C), high-density lipoprotein cholesterol (HDL-C), and fasting plasma glucose (FPG) were determined using enzymatic colorimetric methods. High-sensitivity C-reactive protein (hs-CRP) was assessed via immunoturbidimetric assay on a Hitachi 7180 autoanalyzer (Hitachi, Tokyo, Japan). All biochemical assays demonstrated a coefficient of variation (CV) below 5%, confirming high reproducibility and analytical reliability of the measured parameters.

### Assessment of basal metabolic rate

Although indirect calorimetry remains the gold standard for determining basal metabolic rate (BMR), its application in large-scale epidemiological settings is constrained by high operational demands and cost. As a validated, practical alternative for Chinese population-based studies, we estimated BMR using the Singapore equation [26]. To evaluate the robustness of our primary findings, we also performed a sensitivity analysis by recalculating BMR using the Harris–Benedict and Mifflin–St Jeor equations [27, 28]. The formulas are detailed as follows:

1. Singapore equation:
  Male: BMR (kcal/day) = 0.2389 × [52.6 × weight (kg) + 828 × 1 + 1960];
  Female: BMR (kcal/day) = 0.2389 × [52.6 × weight (kg) + 828 × 0 + 1960].
2. Harris–Benedict equation:
  Male: BMR (kcal/day) = 66.4730 + (13.7516 × weight [kg]) + (5.0033 × height [cm]) − (6.7750 × age [years]);
  Female: BMR (kcal/day) = 665.0955 + (9.5634 × weight [kg]) + (1.8496 × height [cm]) − (4.6756 × age [years]).
3. Mifflin–St Jeor equation:
  Male: BMR (kcal/day) = (9.99 × weight [kg]) + (6.25 × height [cm]) − (4.92 × age [years]) + 5;
  Female: BMR (kcal/day) = (9.99 × weight [kg]) + (6.25 × height [cm]) − (4.92 × age [years]) − 161.

### Calculation of insulin resistance surrogate indices

IR was assessed using three validated surrogate indices derived from routine laboratory and anthropometric measurements [29].The following formulas were applied:

1. TyG = $In$(TG [mg/dl] × FPG [mg/dl]/ 2);      
2. eGDR = 21.158 − 0.09 × WC [cm] − 3.407 × hypertension [yes = 1, no = 0] − 0.551 × HbA1c%;
3. METS-IR = $In$(2 × FPG [mg/dl] + TG [mg/dl]) × BMI [kg/m^2^]/$In$(HDL-C [mg/dl]).    

### Outcome definition and ascertainment

The primary study endpoint was incident CMM, defined as the development of at least two distinct CMDs, including diabetes, heart disease, and stroke [30]. Diabetes mellitus was defined based on standard laboratory and clinical criteria applied to data from the 2011 and 2015 survey waves. The diagnosis required meeting any of the following: FPG ≥ 7.0 mmol/L; glycated hemoglobin (HbA1c) ≥ 6.5%; current use of anti-diabetic medication; or self-report diagnosis (“Have you been diagnosed with diabetes or hyperglycemia by a doctor?”) [31]. Heart disease (including coronary heart disease, angina, or heart failure) and stroke cases were identified through self-reported physician diagnoses or documented use of cardiovascular medications. Participants were specifically asked: “Have you ever been diagnosed with heart disease?” and “Have you ever been diagnosed with a stroke?” The time-to-event was calculated as the interval from the baseline assessment to the follow-up wave in which CMM was first identified. For participants who did not develop CMM during the study period, follow-up time was censored at the date of their last available survey [25].

### Covariates

The analysis incorporated a comprehensive set of covariates, including demographic characteristics, lifestyle factors, and clinical measures. Specifically, we considered age, gender, smoking status, drinking status, education level, marital status, residence, systolic blood pressure (SBP), diastolic blood pressure (DBP), TC, HDL-C, LDL-C, hs-CRP, chronic diseases (including hypertension, dyslipidemia, depression, and cancer), as well as medication use (antihypertensive, antidiabetic, antidyslipidemic, and cardiovascular medications). Education was classified as below primary, primary school, middle school, and high school or above. Marital status was grouped as married or other types (including never married, divorced, separated, or widowed). Smoking and drinking behaviors were recorded dichotomously (yes/no). Residential area was classified as urban or rural. Hypertension was defined by any of the following: self-reported physician diagnosis, SBP ≥ 140 mmHg, DBP ≥ 90 mmHg, or current use of antihypertensive medication [32]. Cancer status was determined based on self-reported diagnosis, history of chemotherapy, or cancer-related surgery. Dyslipidemia was defined as the presence of any of the following: a self-reported history of the condition, current use of lipid-lowering agents, or a lipid profile characterized by TG ≥ 150 mg/dl, TC ≥ 240 mg/dl, HDL-C < 40 mg/dl, or LDL-C ≥ 160 mg/dl [33]. Depression was assessed using the 10-item Center for Epidemiologic Studies Depression Scale (CESD-10) and defined by the standard cut-off score of ≥ 10 [34].

### Handling of missing data

The analysis accounted for incomplete data across multiple study variables. The frequency and proportion of missing values were as follows: cardiovascular medications (n = 1, 0.01%), drinking status (n = 4, 0.06%), antihypertensive medication (n = 5, 0.07%), antidiabetic medication (n = 6, 0.08%), history of cancer (n = 11, 0.15%), LDL-C (n = 12, 0.17%), SBP (n = 52, 0.72%), DBP (n = 53, 0.74%), depression (n = 60, 0.83%), dyslipidemia (n = 90, 1.25%), and antidyslipidemic medication (n = 92, 1.28%). Overall, missing data accounted for 3.19% (230 of 7204) of all data points and were assumed to be missing at random. To minimize potential bias attributable to missing information, we performed multiple imputation using chained equations (MICE) with Markov chain Monte Carlo (MCMC) sampling. Five imputed datasets were generated, and results were pooled according to Rubin's rules to derive final parameter estimates.

### Statistical analysis

Normally distributed continuous variables are expressed as mean (standard deviation, SD), whereas skewed continuous variables are expressed as the median (interquartile range, IQR). Between-group differences were compared using Student's t-tests or Mann–Whitney U tests. Categorical variables are presented as frequencies (percentages), with between-group comparisons performed using chi-square tests or Fisher's exact tests. As established clinical cutoffs for IR surrogate indices and BMR are lacking, these variables were dichotomized at their median values. Given the known sex-based differences in BMR, sex-specific median thresholds were applied to classify participants into high or low BMR subgroups. Subsequently, participants were stratified into four combined exposure groups based on their IR and BMR categories: (1) low IR and low BMR, (2) low IR and high BMR, (3) high IR and low BMR, and (4) high IR and high BMR.

Kaplan–Meier analysis was performed to assess the cumulative incidence of CMM, and the log-rank test was applied to compare differences across groups. Incidence rates of CMM events were reported as per 1000 person-years. The association of IR surrogate indices and BMR with CMM events were analyzed through univariate and multivariate Cox regression models with hazard ratios (HRs) and 95% confidence intervals (CIs) reported. The proportional hazards assumption was confirmed by Schoenfeld residuals. Additionally, multicollinearity diagnostics (Tables S1–S7) revealed all generalized variance inflation factors (GVIFs) were below 5, indicating no significant multicollinearity among covariates. We constructed three sequential Cox proportional hazards models to evaluate the association between exposure groups and incident CMM. Model I was non-adjusted. Model II was adjusted for age, gender, smoking status, drinking status, education level, marital status, residence. Model III was further adjusted for SBP, DBP, TC, HDL-C, LDL-C, hs-CRP, chronic diseases (hypertension, dyslipidemia, depression, and cancer), and medication use (antihypertensive, antidiabetic, antidyslipidemic, and cardiovascular medications). To examine potential nonlinear relationships between IR surrogate indices (TyG, eGDR, and METS-IR) and CMM risk across different BMR strata, we performed restricted cubic spline (RCS) analyses with full adjustment for covariates.

Receiver operating characteristic (ROC) curves were constructed to evaluate the predictive performance of IR surrogate indices and BMR for CMM risk, both individually and in combination. Predictive accuracy was quantified using the area under the curve (AUC). The net reclassification improvement (NRI) and integrated discrimination improvement (IDI) were computed to assess incremental predictive value [35]. Mediation analysis was employed to assess the direct and indirect effects between IR surrogate indices, BMR, and the development of CMM. Specifically, BMR (dichotomized at the median) was defined as the exposure (X), with each IR surrogate index (dichotomized at median) included as a mediator (M), and CMM as the outcome (Y). Reciprocally, the complementary model treated each IR surrogate index as the exposure (X) and BMR as the mediator (M). This analytical approach has been commonly adopted in epidemiological studies to quantify mediation effect [36].

Additive and multiplicative interactions between BMR and IR surrogate indices were evaluated by incorporating their product term in the model. In accordance with methodological recommendations, protective factors require recoding to appropriately evaluate additive interaction measures [37]. We therefore defined high eGDR levels as the reference category to satisfy risk-factor assumptions. Multiplicative interaction was evaluated through the HR (95% CI) of the product term in regression models. Additive interaction was assessed using three established metrics: the relative excess risk due to interaction (RERI), the attributable proportion due to interaction (AP), and the synergy index (SI). All measures were calculated with 95% CIs derived via the delta method.

Subgroup analyses were conducted to evaluate the association between combined BMR and IR surrogate indices and incident CMM, stratified by the following variables: BMI (< 24 vs. ≥ 24 kg/m^2^), age (< 60 vs. ≥ 60 years), gender (male vs. female), smoking status (yes vs. no), drinking status (yes vs. no), hypertension (yes vs. no), dyslipidemia (yes vs. no), depression (yes vs. no), diabetes (yes vs. no), heart disease (yes vs. no), stroke (yes vs. no), antihypertensive medication use (yes vs. no), and antidyslipidemic medication use (yes vs. no). Several sensitivity analyses were also conducted to ensure the robustness of the primary findings. In sensitivity analysis 1, BMR was recalculated using the Harris–Benedict and Mifflin–St Jeor equations, and the joint associations of IR indices and BMR with CMM risk were re-evaluated. In sensitivity analysis 2, we excluded participants diagnosed with CMM during the initial 2 years of follow-up. In sensitivity analysis 3, we employed multiple imputation to address missing data and reduce potential bias due to incomplete information. In sensitivity analysis 4, participants who received treatment for diabetes, heart disease, stroke, hypertension, or dyslipidemia at baseline were excluded. In sensitivity analysis 5, participants with baseline diagnoses of diabetes, heart disease, or stroke were excluded to minimize residual confounding. In sensitivity analysis 6, we examined the associations of BMR and IR indices with the risks of developing diabetes, heart disease, and stroke separately.

All analyses were performed using R statistical software (version 4.4.3), SPSS (version 27.0.1), and GraphPad Prism (version 9.0). Specific analyses were implemented with the following R packages: mediation analysis using the ‘mediation’ package, multiple imputation using the ‘mice’ package, additive interaction assessment using the ‘interactionR’ package, and Cox regression using the ‘survival’ package. The Kaplan–Meier survival curves were generated in GraphPad Prism, and the graphical abstract was created with Figdraw. A two-tailed *P* value < 0.05 was considered statistically significant.

## Results

### Baseline characteristics of study participants

A total of 7204 participants were included in the final analytical cohort and followed from 2011 to 2020. During the maximum 9.0-year follow-up period, 1103 individuals (15.3%) developed CMM. Baseline characteristics stratified by CMM status are presented in Table 1. The overall study population had a median age of 57.0 years (IQR: 51.0–63.0), with 3217 participants (44.7%) being male. Compared to those who did not develop CMM, participants who progressed to CMM were significantly older and exhibited more adverse metabolic profiles, including higher WC, body mass index (BMI), SBP, DBP, TG, TC, LDL-C, FPG, HbA1c, hs-CRP levels, along with lower HDL-C levels. The CMM group also demonstrated significantly higher prevalence rates of hypertension, dyslipidemia, depression, and cancer. Furthermore, participants who developed CMM were more likely to be female, reside in urban areas, and use medications including antihypertensive, antidiabetic, antidyslipidemic, and cardiovascular medications. All IR surrogate indices and BMR measurements differed significantly between groups, with CMM participants showing higher TyG index, METS-IR, and BMR values, but lower eGDR values (all *P* < 0.05).

**Table 1:** Baseline characteristics of the participants classified by CMM

Characteristics	Overall	Non-CMM	CMM	*P* value
	N = 7204	N = 6101	N = 1103	
Age, years	57.00 (51.00, 63.00)	57.00 (51.00, 63.00)	60.00 (55.00, 66.00)	< 0.001
Gender, n (%)				0.023
Male	3217 (44.66)	2759 (45.22)	458 (41.52)	
Female	3987 (55.34)	3342 (54.78)	645 (58.48)	
BMI, kg/m^2^, n (%)				< 0.001
< 24	4245 (58.93)	3753 (61.51)	492 (44.61)	
≥ 24	2959 (41.07)	2348 (38.49)	611 (55.39)	
Education level, n (%)				0.634
Below primary	3413 (47.38)	2873 (47.09)	540 (48.96)	
Primary school	1605 (22.28)	1338 (21.93)	267 (24.21)	
Middle school	1474 (20.46)	1280 (20.98)	194 (17.59)	
High school or above	712 (9.88)	610 (10.00)	102 (9.25)	
Residence, n (%)				0.041
Urban	2381 (33.05)	1987 (32.57)	394 (35.72)	
Rural	4823 (66.95)	4114 (67.43)	709 (64.28)	
Married, n (%)	6140 (85.23)	5232 (85.76)	908 (82.32)	0.053
Smoking, n (%)	2685 (37.27)	2295 (37.62)	390 (35.36)	0.153
Drinking, n (%)	2750 (38.17)	2338 (38.32)	412 (37.35)	0.542
SBP, mmHg	125.50 (113.00, 140.00)	124.00 (112.00, 138.50)	133.00 (119.50, 148.50)	< 0.001
DBP, mmHg	74.00 (66.50, 82.50)	73.50 (66.50, 82.00)	77.50 (69.00, 85.50)	< 0.001
Waist circumference, cm	84.30 (77.60, 91.80)	84.00 (77.00, 90.60)	89.10 (82.00, 96.20)	< 0.001
TC (mg/dl)	190.98 (168.17, 215.72)	190.21 (167.40, 214.56)	197.17 (173.20, 222.68)	< 0.001
TG (mg/dl)	105.32 (75.22, 153.99)	101.78 (73.46, 148.68)	123.01 (87.61, 184.52)	< 0.001
HDL-C (mg/dl)	49.48 (40.59, 59.92)	50.26 (41.37, 60.70)	46.01 (37.50, 56.06)	< 0.001
LDL-C (mg/dl)	115.21 (94.33, 137.63)	114.43 (93.94, 136.47)	119.07 (95.88, 143.43)	0.006
FPG (mg/dl)	102.06 (94.32, 112.14)	101.16 (93.78, 110.16)	108.72 (98.46, 125.55)	< 0.001
HbA1c (%)	5.10 (4.90, 5.40)	5.10 (4.90, 5.40)	5.30 (5.00, 5.65)	< 0.001
hs-CRP (mg/L)	0.97 (0.53, 2.02)	0.92 (0.52, 1.90)	1.30 (0.67, 2.65)	< 0.001
BMR ((kcal)	1288.5 (1148.1, 1427.0)	1285.0 (1142.4, 1420.0)	1307.7 (1173.8, 1469.0)	< 0.001
TyG	8.59 (8.23, 9.02)	8.55 (8.20, 8.98)	8.81 (8.44, 9.31)	< 0.001
eGDR	10.33 (7.79, 11.23)	10.50 (8.38, 11.31)	8.02 (6.43, 10.40)	< 0.001
METS-IR	34.38 (29.78, 40.29)	33.75 (29.45, 39.28)	38.49 (32.69, 44.61)	< 0.001
Basal chronic disease, n (%)				
Hypertension	2215 (30.75)	1630 (26.72)	585 (53.04)	< 0.001
Dyslipidemia	721 (10.01)	481 (7.88)	240 (21.76)	< 0.001
Depression	2660 (36.92)	2118 (34.72)	542 (49.14)	< 0.001
Cancer	75 (1.04)	57 (0.93)	18 (1.63)	0.036
Diabetes	1013 (14.06)	679 (11.13)	334 (30.28)	< 0.001
Heart disease	701 (9.73)	432 (7.08)	269 (24.39)	< 0.001
Stroke	105 (1.46)	55 (0.90)	50 (4.53)	< 0.001
Medications, n (%)				
Antihypertensive	1211 (16.81)	836 (13.70)	375 (34.00)	< 0.001
Antidiabetic	166 (2.30)	93 (1.52)	73 (6.62)	< 0.001
Antidyslipidemic	364 (5.05)	248 (4.06)	116 (10.52)	< 0.001
Cardiovascular medications	376 (5.22)	217 (3.56)	159 (14.42)	< 0.001

Date are presented as median (25th to 75th interquartile range) or n (%)

*CMM* Cardiometabolic multimorbidity, *BMI* Body mass index, *SBP* Systolic blood pressure, *DBP* Diastolic blood pressure, *TC* Total cholesterol, *TG* Triglyceride, *HDL-C* High-density lipoprotein cholesterol, *LDL-C* Low-density lipoprotein cholesterol, *FPG* Fasting plasma glucose, *HbA1c* Glycated hemoglobin, *hs-CRP* High-sensitivity C-reactive protein, *BMR* Basal metabolic rate, *TyG* Triglyceride-glucose, *eGDR* Estimate glucose disposal rate, *METS-IR* Metabolic score for insulin resistance

### Associations of TyG, eGDR, METS-IR and BMR with the risk of developing CMM

**Fig. 2:**
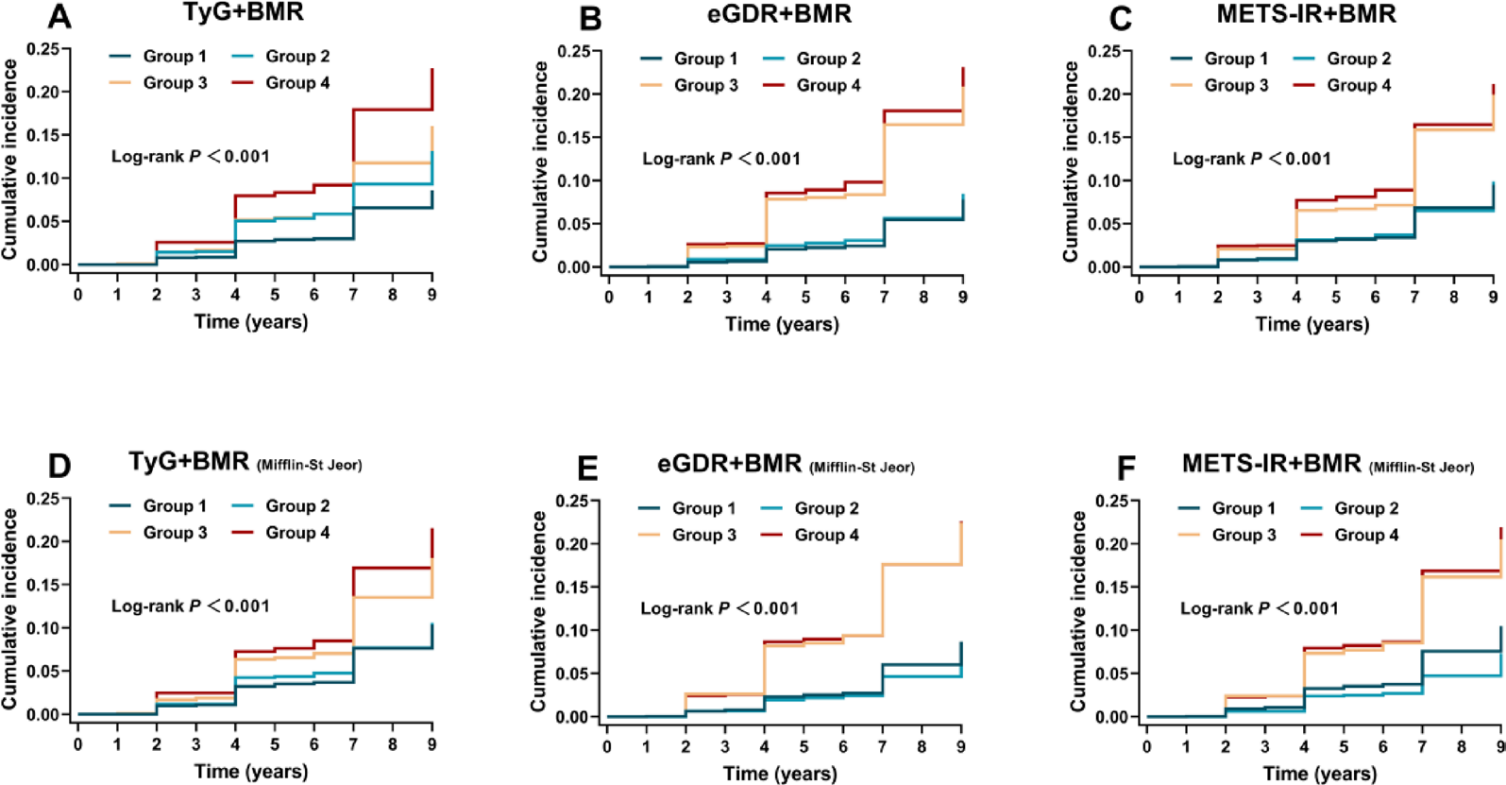
Kaplan–Meier curves for the cumulative incidence of CMM, stratified by levels of IR surrogate indices and BMR (A-F). Group 1 refers to low IR and low BMR; Group 2 refers to low IR and high BMR; Group 3 refers to high IR and low BMR; Group 4 refers to high IR and high BMR. *IR* insulin resistance, *BMR* basal metabolic rate, *TyG* triglyceride-glucose, *eGDR* estimate glucose disposal rate, *METS-IR* metabolic score for insulin resistance

As shown in Table S8, elevated levels of BMR, TyG, and METS-IR were significantly associated with an increased risk of CMM after full adjustment, whereas higher eGDR was associated with a reduced risk. We further evaluated the joint effects of IR indices and BMR on CMM risk. Kaplan–Meier curves demonstrated the highest cumulative incidence among participants with high IR and high BMR, and the lowest incidence among those with low IR and low BMR (all log-rank *P* < 0.001; Fig. 2A–C). Sensitivity analyses confirmed the robustness of these joint associations for TyG and eGDR when BMR was recalculated using the Harris–Benedict and Mifflin–St Jeor equations; however, the association was attenuated for METS-IR (Figs. 2D–F and S1). Cox regression analyses confirmed that participants with combined high IR and high BMR exhibited the greatest CMM risk after full adjustment. Specifically, the combination of high TyG and high BMR was associated with a 2.13-fold increased risk (HR = 2.13, 95% CI 1.73–2.63) compared to the reference group (low TyG and low BMR). Similarly elevated risks were observed for the joint presence of low eGDR and high BMR (HR = 1.93, 95% CI 1.57–2.36) and high METS-IR and high BMR (HR = 1.92, 95% CI 1.61–2.29) (Table 2).

**Table 2:** Combined effects of insulin resistance surrogate indices and basal metabolic rate on CMM risk

Subgroups	Incidence rate^a^	Model I		Model II		Model III	
		HR (95% CI)	*P* value	HR (95% CI)	*P* value	HR (95% CI)	*P* value
TyG and BMR							
Group 1	8.61	Reference	–	Reference	–	Reference	–
Group 2	13.15	1.57 (1.28, 1.93)	< 0.001	1.80 (1.46, 2.21)	< 0.001	1.51 (1.22, 1.87)	< 0.001
Group 3	16.01	1.93 (1.59, 2.36)	< 0.001	1.89 (1.55, 2.30)	< 0.001	1.61 (1.30, 1.99)	< 0.001
Group 4	22.71	2.87 (2.42, 3.41)	< 0.001	3.17 (2.66, 3.78)	< 0.001	2.13 (1.73, 2.63)	< 0.001
*P* for trend			< 0.001		< 0.001		< 0.001
eGDR and BMR							
Group 1	7.81	Reference	–	Reference	–	Reference	–
Group 2	8.46	1.09 (0.84, 1.40)	0.516	1.24 (0.96, 1.61)	0.095	1.17 (0.90, 1.51)	0.236
Group 3	20.88	2.90 (2.38, 3.53)	< 0.001	2.56 (2.10, 3.12)	< 0.001	1.47 (1.16, 1.86)	0.002
Group 4	23.12	3.26 (2.77, 3.83)	< 0.001	3.36 (2.85, 3.97)	< 0.001	1.93 (1.57, 2.36)	0.001
*P* for trend			< 0.001		< 0.001		< 0.001
METS-IR and BMR							
Group 1	9.50	Reference	–	Reference	–	Reference	–
Group 2	9.90	1.04 (0.80, 1.36)	0.755	1.24 (0.95, 1.62)	0.111	1.22 (0.94, 1.60)	0.140
Group 3	19.94	2.23 (1.82, 2.74)	< 0.001	2.22 (1.80, 2.73)	< 0.001	1.81 (1.44, 2.28)	< 0.001
Group 4	21.14	2.39 (2.07, 2.77)	< 0.001	2.68 (2.31, 3.11)	< 0.001	1.92 (1.61, 2.29)	< 0.001
*P* for trend			< 0.001		< 0.001		< 0.001

Model I: non-adjusted

Model II: adjusted for age, gender, smoking status, drinking status, education level, marital status, residence

Model III: further adjusted for SBP, DBP, TC, HDL-C, LDL-C, hs-CRP, chronic diseases (hypertension, dyslipidemia, depression, and cancer), and medication use (antihypertensive, antidiabetic, antidyslipidemic, and cardiovascular medications)

Group 1 refers to low IR and low BMR; Group 2 refers to low IR and high BMR; Group 3 refers to high IR and low BMR; Group 4 refers to high IR and high BMR. *IR* Insulin resistance, *BMR* Basal metabolic rate, *TyG* Triglyceride-glucose, *eGDR* Estimate glucose disposal rate, *METS-IR* Metabolic score for insulin resistance, *HR* Hazard ratio, *CI* Confidence interval

^a^Incident rate was presented as per 1000 person-years of follow-up

### The detection of nonlinear relationships

**Fig. 3:**
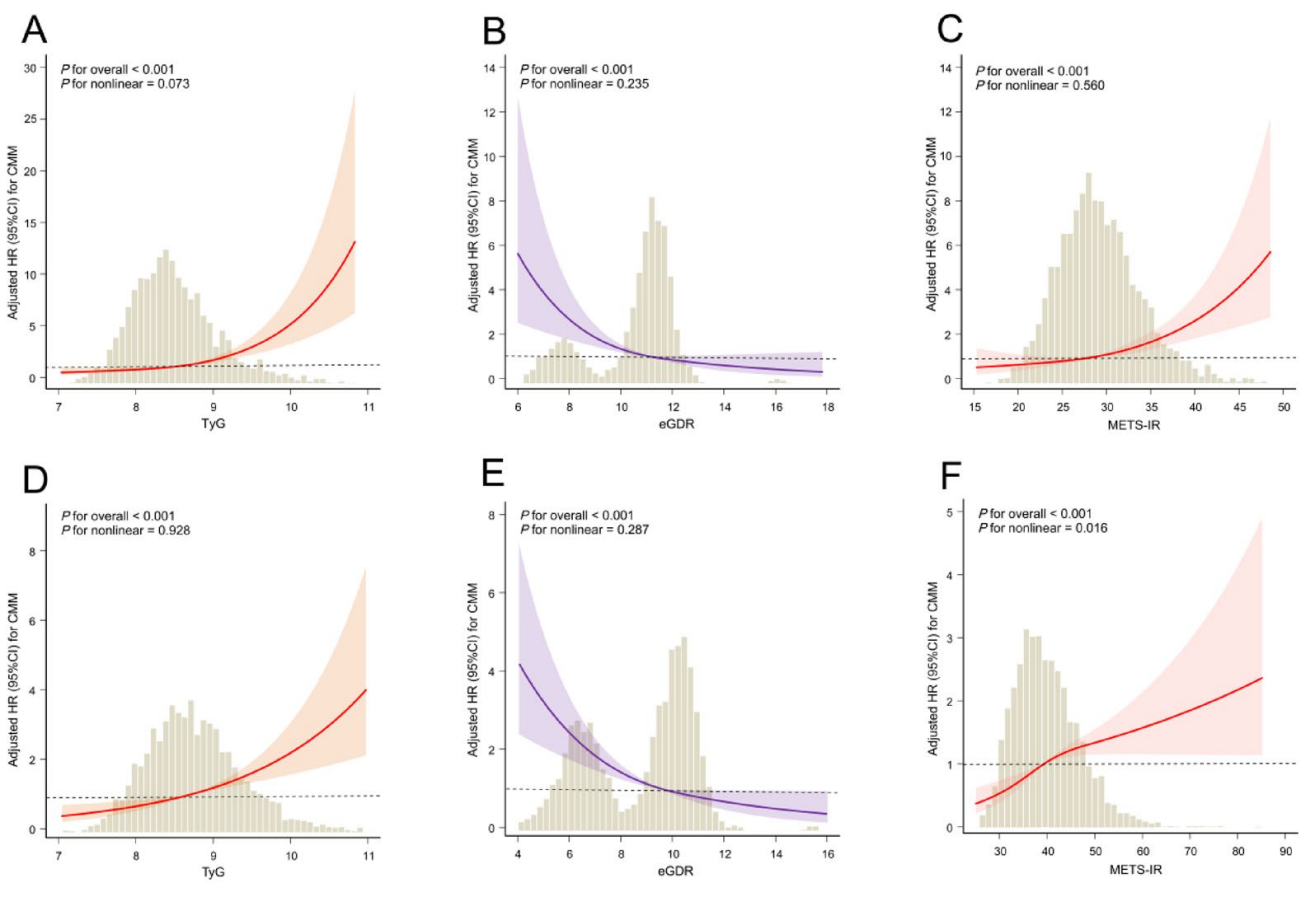
Dose–response relationships of IR surrogate indices (TyG, eGDR, and METS-IR) with CMM risk by BMR strata. Spline analyses were adjusted for age, gender, smoking status, drinking status, education level, marital status, residence, SBP, DBP, TC, HDL-C, LDL-C, hs-CRP, chronic diseases (hypertension, dyslipidemia, depression, and cancer), and medication use (antihypertensive, antidiabetic, antidyslipidemic, and cardiovascular medications). *IR* insulin resistance, *BMR* basal metabolic rate, *TyG* triglyceride-glucose, *eGDR* estimate glucose disposal rate, *METS-IR* metabolic score for insulin resistance, *HR* hazard ratio, *CI* confidence interval

To evaluate potential nonlinear associations, we conducted multivariable-adjusted RCS analyses to model the relationship between IR surrogate indices (TyG, eGDR, METS-IR) and CMM risk, with stratification by BMR. In the low BMR stratum, significant linear dose–response relationships were observed. Specifically, both TyG and METS-IR exhibited positive linear associations with CMM risk, whereas eGDR showed an inverse linear association (All models: *P* for overall < 0.05 and *P* for nonlinear > 0.05; Fig. 3A–C). Among participants with high BMR, the positive linear association for TyG and the inverse linear association for eGDR remained statistically significant (*P* for overall < 0.05 and *P* for nonlinear > 0.05; Fig. 3D–F). In contrast, the association between METS-IR and CMM risk in the high BMR subgroup demonstrated a significant nonlinear relationship (*P* for nonlinear = 0.016). To quantify this nonlinear association, we we performed a two-piecewise linear regression within the Cox proportional hazards model. In the fully adjusted model, a significant inflection point was identified for METS-IR at 51.90 (*P* for log-likelihood ratio test < 0.001; Table S9). Below this threshold, each unit increase in METS-IR was associated with a significantly elevated hazard for CMM (HR = 1.05, 95% CI 1.03–1.07). Conversely, above this value, no significant association was observed (HR = 1.00, 95% CI 0.99–1.01).

### Predictive performance of IR surrogates and BMR for CMM

**Fig. 4:**
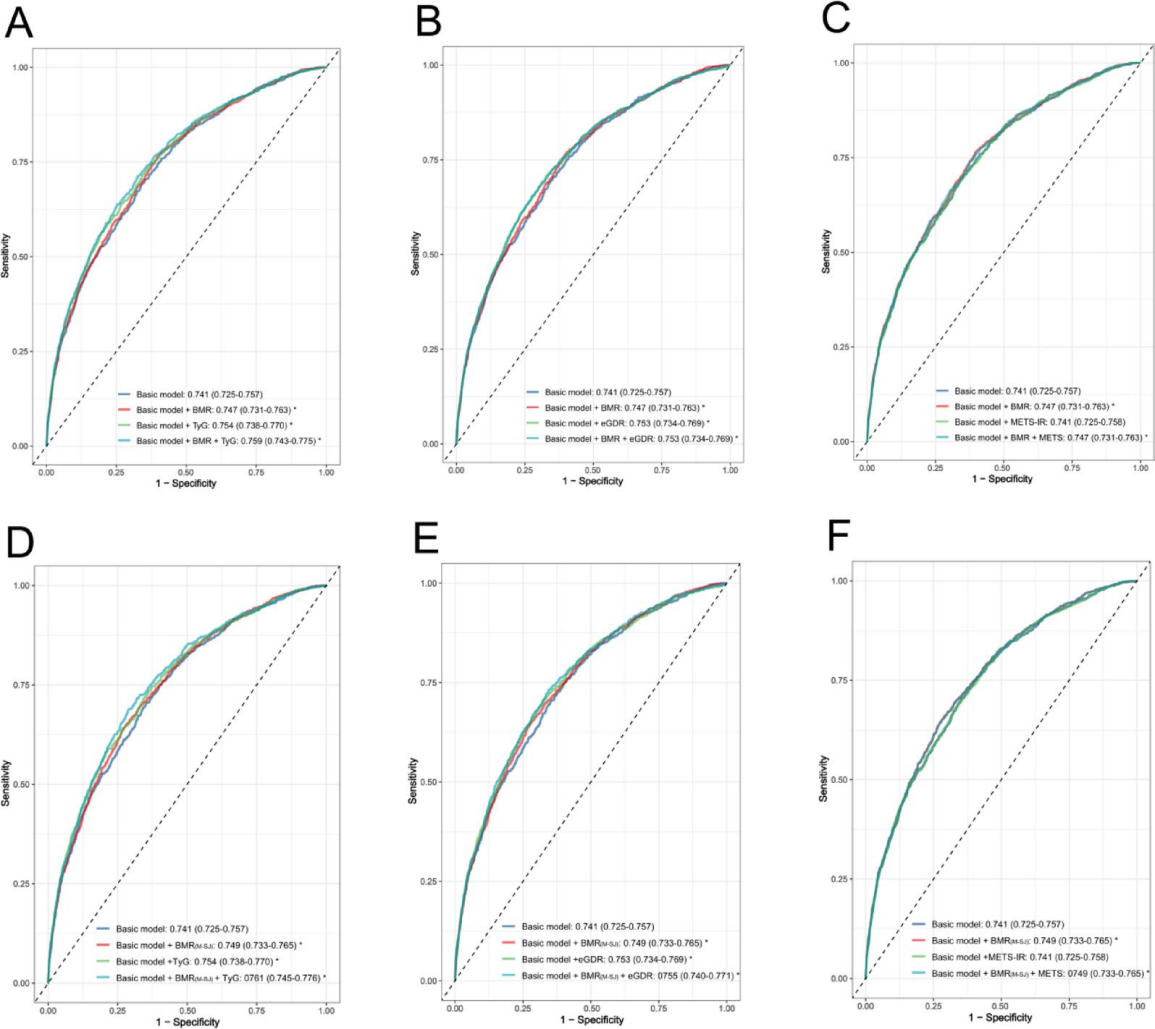
ROC curves for CMM prediction, stratified by BMR assessment method. The curves evaluate insulin resistance surrogates (TyG, eGDR, METS-IR) alone and combined with BMR. Panels A-C and D-F show the corresponding results for the Singapore equation and Mifflin-St Jeor equation, respectively. The basic model was adjusted for age, gender, smoking status, drinking status, education level, marital status, residence, SBP, DBP, TC, HDL-C, LDL-C, hs-CRP, chronic diseases (hypertension, dyslipidemia, depression, and cancer), and medication use (antihypertensive, antidiabetic, antidyslipidemic, and cardiovascular medications). * indicates significantly improved predictive performance versus the basic model by DeLong's test (*P* < 0.05)

The predictive performance of IR surrogates and BMR for assessing CMM risk was evaluated using ROC curves, NRI, and IDI (Figs. 4, S2; Tables S10–S12). When assessed individually, the TyG index showed the most significant AUC improvement when added to the basic model, increasing from 0.741 to 0.754 (*P* < 0.001, DeLong's test). In contrast, the eGDR showed superior reclassification capacity (NRI = 0.342, 95% CI 0.277–0.406). The TyG index also provided the highest discrimination gain (IDI = 0.014, 95% CI 0.010–0.019). In combined models, the integration of BMR with the TyG index yielded optimal overall predictive performance, achieving the highest AUC (0.759, 95% CI 0.743–0.775), along with significant NRI (0.371, 95% CI 0.306–0.436) and IDI (0.017, 95% CI 0.012–0.022). These findings indicate that IR surrogates, particularly in combination with BMR, enhance CMM risk prediction beyond conventional risk factors. However, sensitivity analyses using BMR values derived from the Harris-Benedict and Mifflin–St Jeor equations revealed a decline in predictive performance when these estimates were combined with IR surrogates. Despite this reduction, both NRI and IDI continued to demonstrate statistically significant incremental predictive value compared to the basic model.

### Mediation and interaction analyses of IR surrogates and BMR on CMM risk

**Fig. 5:**
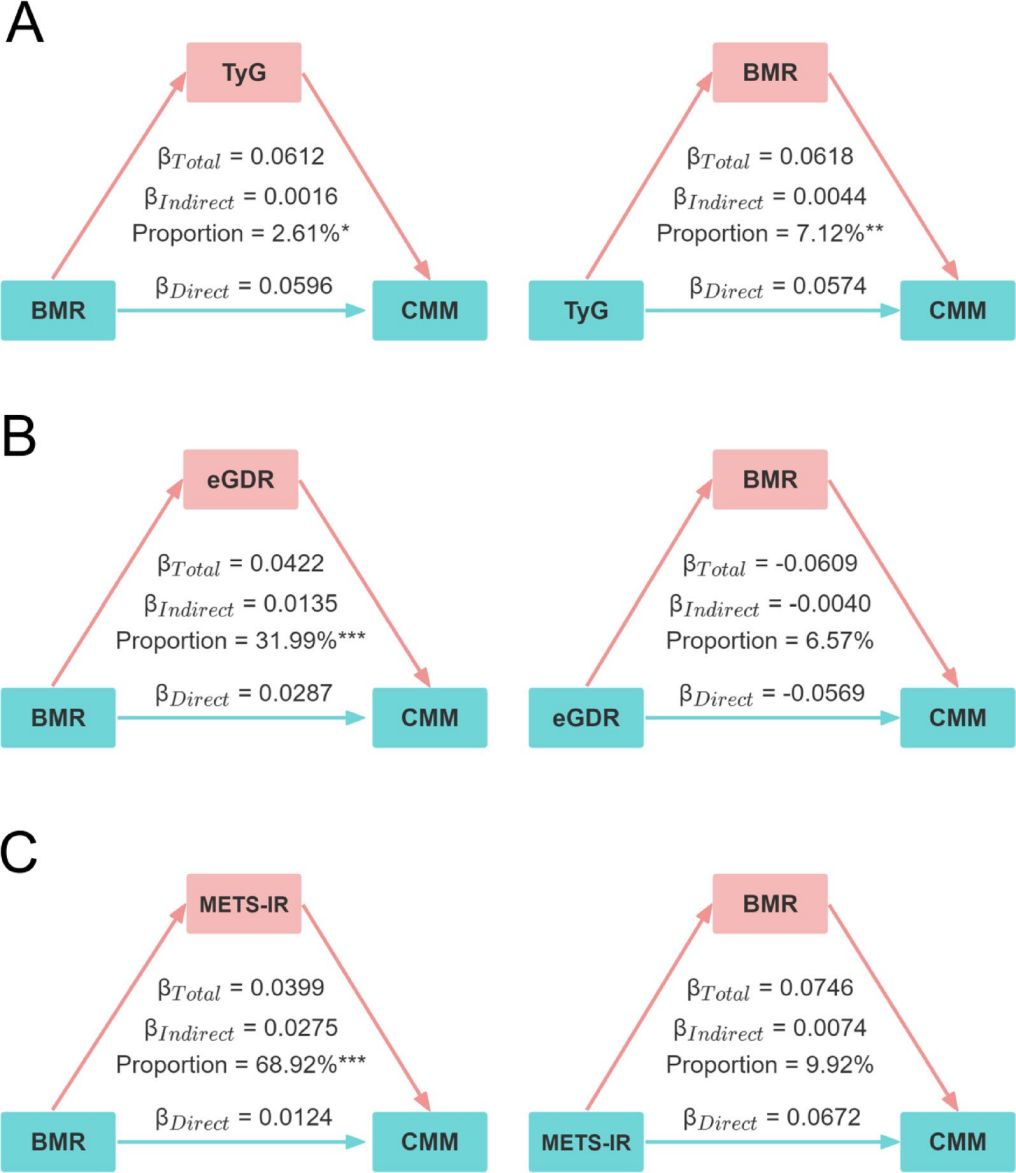
Bidirectional mediation analysis of the TyG index (**A**), eGDR (**B**), METS-IR (**C**), and BMR on CMM risk. Adjusted for age, gender, smoking status, drinking status, education level, marital status, residence, SBP, DBP, TC, HDL-C, LDL-C, hs-CRP, chronic diseases (hypertension, dyslipidemia, depression, and cancer), and medication use (antihypertensive, antidiabetic, antidyslipidemic, and cardiovascular medications). *P < 0.05; **P < 0.01; ***P < 0.001

Mediation analyses were performed to examine the potential bidirectional mediating effects between IR surrogate indices (TyG, eGDR, and METS-IR) and BMR in relation to the risk of CMM (Fig. 5). After full adjustment for covariates, TyG, eGDR, and METS-IR significantly mediated the association between BMR and CMM risk, with proportion mediated values of 2.61%, 31.99%, and 68.92%, respectively. Conversely, BMR significantly mediated the relationship between TyG and CMM, explaining 7.12% of the effect. In contrast, BMR did not exhibit a significant mediation effect in the associations of eGDR or METS-IR with CMM risk (all *P* > 0.05). Collectively, these findings suggest that the impact of BMR on CMM is largely mediated by insulin resistance, with TyG, eGDR, and METS-IR serving as important surrogate indices in this pathway. Interaction analyses were conducted to evaluate potential additive and multiplicative effects between IR surrogate indices and BMR on CMM risk (Table S13). After full adjustment, no significant interactions were observed for any IR surrogate. On the additive scale, the 95% CIs for the RERI and AP included 0. On the multiplicative scale, the 95% CIs for the synergy index (SI) and the interaction term included 1.

### Subgroup and sensitivity analyses

**Fig. 6:**
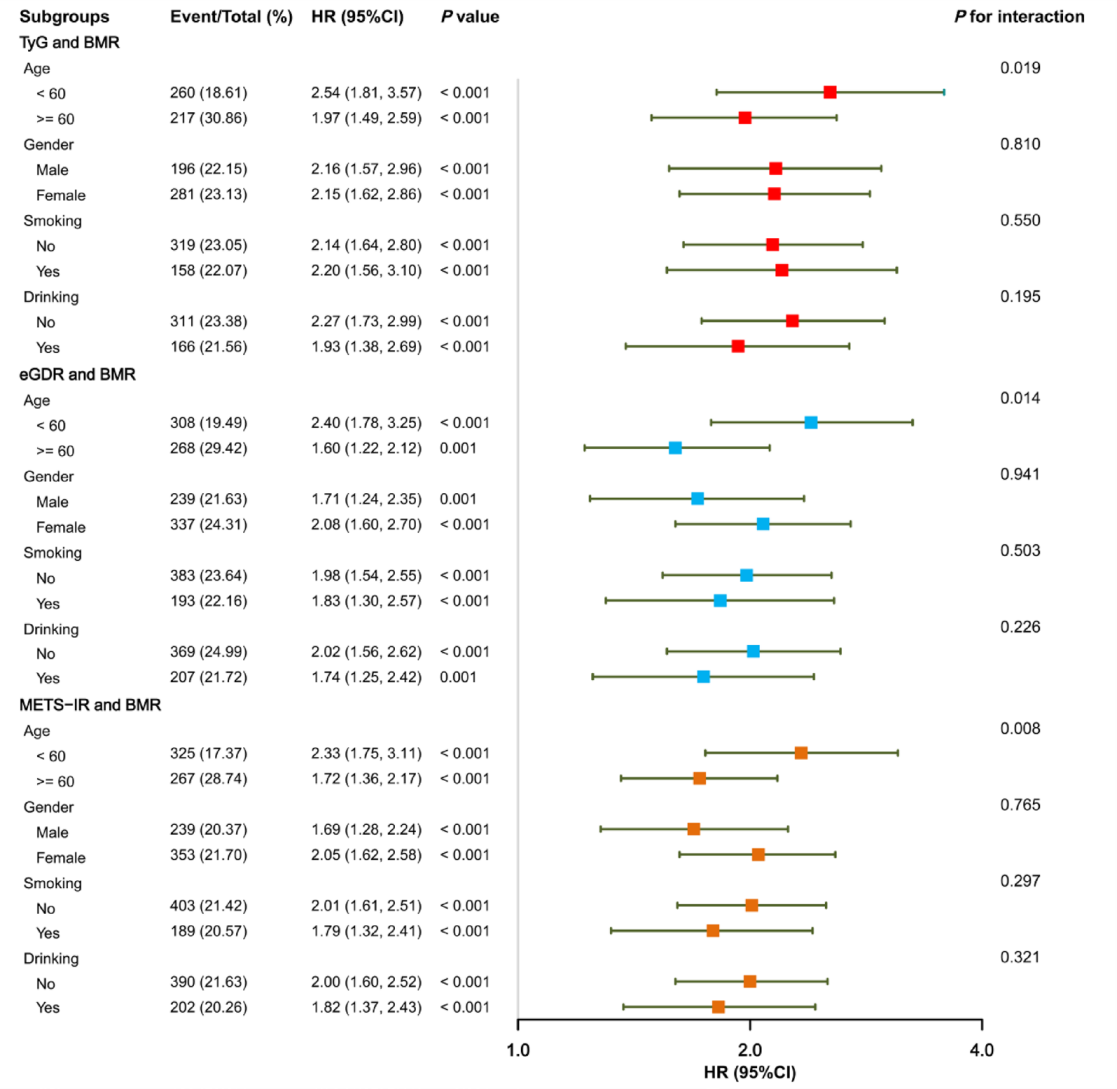
Forest plot of multivariable-adjusted Cox regression analyses in the subgroup of participants with both high IR and high BMR (A–F). Multivariate models were adjusted for age, gender, smoking status, drinking status, education level, marital status, residence, SBP, DBP, TC, HDL-C, LDL-C, hs-CRP, chronic diseases (hypertension, dyslipidemia, depression, and cancer), and medication use (antihypertensive, antidiabetic, antidyslipidemic, and cardiovascular medications), with the exception of the stratification variable

Subgroup analyses were performed to further evaluate the combined effect of high IR and high BMR with CMM risk across the following strata: BMI, age, gender, smoking status, drinking status, chronic conditions (including hypertension, dyslipidemia, diabetes, heart disease, and stroke), and medication use (antihypertensive and antidyslipidemic agents) (Figs. 6 and S3–S5). After full adjustment for covariates, no significant interactions were detected by BMI, gender, smoking status, drinking status, chronic conditions (including hypertension, dyslipidemia, diabetes, heart disease, and stroke), and medication use (antihypertensive and antidyslipidemic agents) (*P* for interaction > 0.05). In contrast, a significant interaction was identified for age (*P* for interaction < 0.05), with the combined exposure conferring a higher CMM risk among younger participants. To verify the robustness of the primary findings, several sensitivity analyses were conducted. These included recalculating BMR using the Harris–Benedict and Mifflin–St Jeor equations (n = 6974), excluding participants with incomplete data (n = 6974), excluded participants who developed CMM within the first two years of follow-up (n = 6870), removing those receiving treatment for diabetes, cardiovascular, hypertension, or dyslipidemia at baseline (n = 5439), excluding participants with baseline diagnoses of diabetes, heart disease, or stroke (n = 5237), and examined the associations of BMR and IR indices with the risks of incident diabetes, heart disease, and stroke separately (n = 5237). Importantly, all analyses yielded results consistent with the main findings (Table 3 and Tables S14–S19).

**Table 3:** Sensitivity analysis of the joint effects of IR surrogate indices and BMR on the risks of developing diabetes, heart disease, and stroke, respectively (n** = **5237)

Subgroups	Diabetes^b^		*P* value	Heart disease^b^		*P* value	Stroke^b^		*P* value
	Incidence rate^a^	HR (95% CI)		Incidence rate^a^	HR (95% CI)		Incidence rate^a^	HR (95% CI)	
TyG and BMR									
Group 1	12.26	Reference	–	15.55	Reference	–	5.81	Reference	–
Group 2	14.59	1.08 (0.86, 1.36)	0.483	20.11	1.37 (1.13, 1.66)	0.117	9.26	1.47 (1.07, 2.02)	0.016
Group 3	15.70	1.24 (1.00, 1.54)	0.055	18.32	1.18 (0.96, 1.45)	0.001	10.14	1.59 (1.18, 2.14)	0.002
Group 4	24.61	1.75 (1.41, 2.18)	< 0.001	22.29	1.52 (1.24, 1.87)	< 0.001	10.75	1.62 (1.20, 2.20)	0.002
*P* for trend			< 0.001			< 0.001			0.034
eGDR and BMR									
Group 1	12.56	Reference	–	14.90	Reference	–	5.84	Reference	–
Group 2	12.16	1.02 (0.80, 1.32)	0.855	16.74	1.24 (0.99, 1.55)	0.057	4.58	0.84 (0.57, 1.26)	0.407
Group 3	16.69	1.07 (0.83, 1.37)	0.614	21.54	1.05 (0.83, 1.33)	0.662	13.27	1.43 (1.03, 2.00)	0.032
Group 4	23.57	1.68 (1.40, 2.03)	< 0.001	23.10	1.41 (1.18, 1.70)	< 0.001	11.68	1.55 (1.18, 2.03)	0.002
*P* for trend			< 0.001			< 0.001			0.001
METS-IR and BMR									
Group 1	12.44	Reference	–	16.70	Reference	–	7.08	Reference	–
Group 2	12.12	1.09 (0.82, 1.43)	0.575	17.23	1.14 (0.90, 1.44)	0.289	7.01	1.12 (0.78, 1.62)	0.547
Group 3	18.49	1.34 (1.04, 1.74)	0.024	16.60	1.01 (0.78, 1.30)	0.968	10.75	1.28 (0.91, 1.80)	0.162
Group 4	22.64	1.78 (1.48, 2.13)	< 0.001	22.45	1.42 (1.19, 1.68)	< 0.001	10.48	1.30 (1.01, 1.68)	0.046
*P* for trend			< 0.001			< 0.001			0.046

Group 1 refers to low IR and low BMR; Group 2 refers to low IR and high BMR; Group 3 refers to high IR and low BMR; Group 4 refers to high IR and high BMR. *IR* Insulin resistance, *BMR* Basal metabolic rate, *TyG* Triglyceride-glucose, *eGDR* Estimate glucose disposal rate, *METS-IR* Metabolic score for insulin resistance, *HR* Hazard ratio, *CI* Confidence interval

^a^Incident rate was presented as per 1000 person-years of follow-up

^b^Multivariable-adjusted for age, gender, smoking status, drinking status, education level, marital status, residence, SBP, DBP, TC, HDL-C, LDL-C, hs-CRP, chronic diseases (hypertension, dyslipidemia, depression, and cancer), and medication use (antihypertensive and antidyslipidemic)

## Discussion

In this national cohort of 7204 middle-aged and older Chinese adults, elevated IR and BMR were independently and jointly associated with an increased risk of CMM. ROC analysis showed that adding IR surrogates to the baseline model significantly improved CMM prediction, with further improvement when BMR was included, supported by NRI and IDI. Mediation analysis indicated that all IR surrogates significantly mediated the relationship between BMR and CMM, while BMR only mediated the association involving the TyG index. No significant additive or multiplicative interactions were found between IR surrogates and BMR on CMM risk. These results suggest that BMR influences CMM primarily through insulin resistance pathways. Combined assessment of IR surrogates and BMR may thus enhance early identification and prevention of CMM.

The increasing global burden of population aging is particularly evident in the rising incidence and mortality of cardiovascular diseases. IR, defined as the impaired ability of insulin to regulate glucose metabolism in key tissues such as skeletal muscle, adipose tissue, and the liver [23], represents one of the earliest pathophysiological abnormalities in the development of cardiometabolic disorders and is a well-established precursor to type 2 diabetes and cardiovascular disease (CVD). Convenient and reliable surrogate indices of IR, including the TyG index, eGDR, and METS-IR, have been widely validated as predictors of CMDs such as diabetes, heart disease, and stroke. In support of this, a study in a middle-aged and older Chinese population reported a significant association between the TyG index and new-onset CVD [38]. Similarly, evidence from large prospective cohorts in Europe and Asia demonstrated that lower cumulative eGDR levels were associated with a higher risk of incident CVD, showing a negative linear dose–response relationship [39]. Another study further indicated that elevated cumulative METS-IR was linked to an increased risk of incident stroke in middle-aged and older Chinese adults [40]. Consistent with this body of evidence, our findings confirm that higher IR levels are significantly associated with an increased risk of CMM [41–43]. However, some studies suggest that the TG/HDL-C ratio may be superior to the TyG index for predicting CMDs [44, 45]. Therefore, we compared the TG/HDL-C ratio with the TyG index, eGDR, and METS-IR to evaluate their value in predicting CMM. Our results demonstrated that the TyG index, eGDR, and METS-IR offered significantly better predictive value (all *P* < 0.001; Fig. S6). Consequently, we focused our subsequent analyses on these three indices.

BMR represents the daily energy expenditure required to maintain essential physiological functions in an awake individual under steady-state conditions [27]. Although indirect calorimetry remains the gold standard for BMR assessment, its application in large-scale epidemiological studies is constrained by high costs and operational complexity [46, 47]. As a practical alternative, predictive equations are widely employed. Previous studies have suggested that elevated BMR may be associated with an increased risk of mortality, particularly from cardiovascular causes [48]. Furthermore, the European Prospective Investigation into Cancer and Nutrition (EPIC) study indicated that higher BMR might serve as a predictor of cancer risk, independent of body fatness [49]. While the relationship between BMR and individual CMDs has been well established [15, 16, 50], its association with CMM and the underlying mechanisms remain unclear. In the present study, BMR was estimated using the Singapore equation, which has been validated as the most accurate predictor in Chinese populations [26]. Consistent with previous findings, our study found a significant association between a higher BMR and an increased risk of CMM (HR = 1.42, 95% CI 1.23–1.63) [16, 17]. Furthermore, we further confirmed that an elevated BMR is significantly associated with an increased risk of diabetes, although this finding appears to contradict prior genetic evidence. [51]. We also investigated the dose–response relationship between BMR and CMM risk. A significant linear positive association was observed in the overall population, while a notable non-linear relationship was identified specifically in females (Fig. S7). Analysis revealed a significant inflection point at a BMR of 1480.47 (*P* for log-likelihood ratio test < 0.001; Table S20). Below this threshold, each unit increase in BMR was associated with a significantly elevated CMM risk (HR = 1.002, 95% CI 1.001–1.003). Conversely, above this value, higher BMR was associated with a significant risk reduction (HR = 0.979, 95% CI 0.963–0.996). These nonlinear trends highlight the importance of developing tailored prevention strategies based on individual metabolic profiles.

Although the association between energy metabolism and cardiovascular disease is well established [52, 53], the specific relationship between BMR and cardiovascular diseases, along with its underlying mechanisms, remains incompletely elucidated. In the present study, we further investigated the joint effects of BMR and IR on CMM risk. Our results showed that participants with concurrently high levels of both IR and BMR faced the highest risk of developing CMM. The robustness of this finding was consistently supported across subgroup and sensitivity analyses. Furthermore, ROC curve assessment revealed that adding IR surrogates to the baseline model substantially improved CMM risk stratification, and the inclusion of BMR provided additional predictive gain, which was similarly supported by the NRI and IDI. Mediation analysis revealed that IR surrogates significantly mediated the association between BMR and CMM risk, with proportion mediated values of 31.99% for eGDR and 68.92% for METS-IR. In contrast, BMR did not significantly mediate the associations of eGDR or METS-IR with CMM. Furthermore, no significant additive or multiplicative interactions were detected between the IR surrogates and BMR on CMM risk. These findings collectively suggest that the effect of BMR on CMM is substantially mediated through insulin resistance pathways, implying that elevated energy expenditure may contribute to CMM pathogenesis partly by exacerbating an insulin-resistant state. This interpretation is corroborated by external evidence. A large longitudinal cohort study confirmed that 6.95% of the relationship between BMR and incident metabolic dysfunction-associated steatotic liver disease (MASLD) was mediated by IR [54]. Similarly, the REACTION study involving 36,115 Chinese adults aged ≥ 40 demonstrated an independent positive association between BMR and IR in the general Chinese population, particularly among women [24]. These convergent results strengthen the hypothesis that an elevated BMR may promote the development of IR, thereby increasing the risk of CMDs in humans.

IR is known to significantly increase the risk of inflammation, atherosclerosis, endothelial dysfunction, and oxidative stress, all of which contribute to the development of CMDs [55, 56]. In the present study, we demonstrated that the effect of BMR on CMM risk is largely mediated through IR pathways. Nevertheless, the association between BMR and IR, as well as its underlying mechanisms, remains incompletely elucidated. BMR, which represents 60–70% of total daily energy expenditure, plays a central role in overall energy homeostasis. Dysregulated energy metabolism, which is often manifested as an elevated BMR, has been implicated in the induction of inflammatory responses, disturbances in glucose metabolism, and mitochondrial dysfunction, all of which may accelerate the progression of CMDs [57, 58]. Moreover, obesity promotes mitochondrial fragmentation and functional impairment in white adipocytes, further aggravating systemic metabolic dysregulation [59]. These conditions, characterized by chronic inflammation and mitochondrial dysfunction, are recognized key contributors to the development of IR [23, 60]. Other studies have found that impaired glucose regulation (IGR) has been significantly associated with both elevated BMR and reduced insulin sensitivity, underscoring a close relationship between heightened energy expenditure and glucose metabolic impairment in adults [24]. To further explore this association in our cohort, we stratified participants according to BMR quartiles. Subsequent analyses revealed a significant correlation between BMR and surrogate indices of IR, showing a graded increase in IR levels across progressively higher BMR subgroups (Fig. S8). We also evaluated other cardiometabolic parameters, including FPG, glycated hemoglobin (HbA1c), CRP, and the Chinese Visceral Adiposity Index (CVAI). The CVAI is a reliable indicator for evaluating visceral fat distribution in the Chinese population and has been widely used to predict the risk of various cardiometabolic diseases [61, 62]. These analyses consistently demonstrated positive correlations between BMR and FPG, HbA1c, CVAI, and CRP (Fig. S9). In addition, the prevalence of abdominal obesity rose significantly with increasing BMR levels (Fig. S10). Collectively, these findings suggest that the chronic inflammation and adipose tissue accumulation associated with IR may trigger an increase in energy expenditure that is compensatory in nature yet ultimately pathological. Thus, a elevated BMR could be interpreted as a clinical indicator of excessive metabolic load and compensatory overdrive. In summary, our study provides the first evidence that elevated IR and BMR act both independently and synergistically to increase the risk of CMM. These insights suggest that future preventive strategies, whether through lifestyle intervention or pharmacotherapy, should not only aim to improve insulin sensitivity but also consider the stabilization of energy metabolism as a potential target for more effective CMM prevention.

This study has several notable strengths. It represents the first systematic evaluation of the joint effect of BMR and surrogate indices of IR on the risk of CMM. Our findings highlight the potential utility of integrating BMR and IR surrogate indices into strategies for the primary prevention of CMM. Secondly, the analysis was conducted using data from a large-scale, prospective, nationwide cohort spanning 28 provinces in China, which provides robust evidence for the associations of BMR and IR with CMM risk. Finally, we are the first to demonstrate that the effect of BMR on CMM risk is substantially mediated through IR pathways. This novel insight offers an important epidemiological foundation for future research into the mechanisms linking energy metabolism, insulin resistance, and cardiometabolic diseases.

Several limitations of this study should be acknowledged. First, IR surrogate indices and BMR were estimated using predictive equations rather than gold-standard methods (such as the hyperinsulinemic-euglycemic clamp and indirect calorimetry), potentially introducing measurement inaccuracy. Nonetheless, these well-validated formulas have been widely employed in large-scale epidemiological investigations due to their operational feasibility and demonstrated utility [26, 63]. Second, in the absence of well-defined clinical thresholds for these parameters, a median-based stratification was employed, which may not represent the optimal threshold for risk discrimination. Third, despite extensive adjustment for confounders in multivariate and subgroup analyses, residual confounding from unmeasured variables (e.g., dietary patterns, physical activity, and socioeconomic factors) cannot be excluded. Fourth, nearly half of the participants were excluded due to missing data, which might lead to a substantial underestimation of CMM incidence. Furthermore, the identification of participants with diabetes, heart disease, or stroke was primarily based on self-reports, which may introduce misclassification bias owing to underreporting or recall inaccuracies. Fifth, since our study population was restricted to middle-aged and older Chinese adults, the generalizability of the findings to other ethnicities, age groups, or populations with different lifestyle characteristics requires further investigation. Finally, given the observational design of this study, we cannot establish a causal relationship between the IR surrogate indices and BMR and the risk of developing CMM. Future prospective studies should employ gold-standard methods such as indirect calorimetry to measure BMR and include more frequent assessments of insulin resistance to better delineate the temporal sequence of changes in these parameters. Furthermore, Mendelian randomization studies utilizing large-scale genetic data are needed to validate the causal nature of the relationships hypothesized in the current study.

## Conclusion

In this large prospective cohort, elevated levels of IR surrogates indices and BMR were independently and jointly associated with an increased risk of CMM. Although no significant interaction between IR surrogates and BMR was observed, mediation analysis demonstrated that the impact of BMR on CMM is substantially mediated by the IR pathway. These findings support an actionable primary prevention strategy: integrating assessments of IR and BMR into routine clinical practice may facilitate earlier identification of high-risk individuals. Such an approach could inform targeted interventions aimed at mitigating the growing burden of CMM by concurrently addressing energy metabolism and insulin sensitivity.

## Supplementary Information

Below is the link to the electronic supplementary material.

Supplementary Material 1

## Data Availability

The datasets analyzed in this study are publicly available from the China Health and Retirement Longitudinal Study (CHARLS). Researchers can access the data by registering and submitting an application via the official CHARLS website: http://charls.pku.edu.cn.

## Abbreviations

TyG: Triglyceride-glucose index
eGDR: Estimated glucose disposal rate
METS-IR: Metabolic score for insulin resistance
BMR: Basal metabolic rate
IR: Insulin resistance
CMDs: Cardiometabolic diseases
CMM: Cardiometabolic multimorbidity
CHARLS: China Health and Retirement Longitudinal Study
RCS: Restricted cubic splines
ROC: Receiver operating characteristic curve
NRI: Net reclassification improvement
IDI: Integrated discrimination improvement
ROS: Reactive oxygen species
WC: Waist circumference
TC: Total cholesterol
TG: Triglyceride
HDL-C: High-density lipoprotein cholesterol
LDL-C: Low-density lipoprotein cholesterol
HbA1c: Glycosylated hemoglobin A1c
FPG: Fasting plasma glucose
SBP: Systolic blood pressure
DBP: Diastolic blood pressure
hs-CRP: High-sensitivity C-reactive protein
IQR: Interquartile range
GVIF: Generalized Variance Inflation Factor
RERI: Relative excess risk due to interaction
AP: Attributable proportion
SI: Synergy index
BMI: Body mass index
HR: Hazard ratio
95% CI: 95% Confidence interval
CVD: Cardiovascular disease
