# Supplementary material for: Joint assessment of insulin resistance surrogate indices and basal metabolic rate for primary prevention of cardiometabolic multimorbidity: evidence from the China Health and Retirement Longitudinal Study (2011–2020)
Source: Cardiovasc Diabetol. 2026 Jan 12;25:37. doi: 10.1186/s12933-025-03067-y (PMC12888234; doi:10.1186/s12933-025-03067-y)
Supplement: Supplementary file 1 — Supplementary Material 1 [file 12933_2025_3067_MOESM1_ESM.docx]

**Table S1** Multicollinearity analysis in the association analysis between BMR and CMM.

| **Variables** | **GVIF** | **Df** | **GVIF^(1/(2*Df))** |
| --- | --- | --- | --- |
| BMR | 1.288 | 1 | 1.135 |
| Age | 1.467 | 1 | 1.211 |
| Sex | 3.198 | 1 | 1.788 |
| Smoking status | 1.856 | 1 | 1.362 |
| Drinking status | 1.490 | 1 | 1.221 |
| Education level | 1.381 | 3 | 1.055 |
| Marital status | 1.100 | 1 | 1.049 |
| Residence | 1.113 | 1 | 1.055 |
| SBP | 2.546 | 1 | 1.596 |
| DBP | 2.514 | 1 | 1.586 |
| TC | 3.140 | 1 | 1.772 |
| HDL-C | 1.200 | 1 | 1.096 |
| LDL-C | 3.080 | 1 | 1.755 |
| hs-CRP | 1.013 | 1 | 1.007 |
| Hypertension | 2.484 | 1 | 1.576 |
| Dyslipidemia | 1.864 | 1 | 1.365 |
| Depression | 1.103 | 1 | 1.050 |
| Cancer | 1.037 | 1 | 1.018 |
| Antihypertensive medication | 2.071 | 1 | 1.439 |
| Antidiabetic medication | 1.131 | 1 | 1.063 |
| Antidyslipidemic medication | 1.790 | 1 | 1.338 |
| Cardiovascular medications | 1.139 | 1 | 1.067 |

Note: Variables with a GVIF greater than 5 or with an adjusted GVIF (GVIF^(1/(2*Df))) greater than 2 were considered collinear and were excluded from the multiple regression model

**Table S2** Multicollinearity analysis in the association analysis between TyG and CMM.

| Variables | GVIF | Df | GVIF^(1/(2*Df)) |
| --- | --- | --- | --- |
| TyG | 1.469 | 1 | 1.212 |
| Age | 1.410 | 1 | 1.188 |
| Sex | 2.318 | 1 | 1.522 |
| Smoking status | 1.847 | 1 | 1.359 |
| Drinking status | 1.478 | 1 | 1.216 |
| Education level | 1.374 | 3 | 1.054 |
| Marital status | 1.098 | 1 | 1.048 |
| Residence | 1.102 | 1 | 1.050 |
| SBP | 2.579 | 1 | 1.606 |
| DBP | 2.521 | 1 | 1.588 |
| TC | 3.806 | 1 | 1.951 |
| HDL-C | 1.425 | 1 | 1.194 |
| LDL-C | 3.432 | 1 | 1.853 |
| hs-CRP | 1.012 | 1 | 1.006 |
| Hypertension | 2.464 | 1 | 1.570 |
| Dyslipidemia | 1.844 | 1 | 1.358 |
| Depression | 1.096 | 1 | 1.047 |
| Cancer | 1.037 | 1 | 1.018 |
| Antihypertensive medication | 2.079 | 1 | 1.442 |
| Antidiabetic medication | 1.114 | 1 | 1.055 |
| Antidyslipidemic medication | 1.784 | 1 | 1.336 |
| Cardiovascular medications | 1.135 | 1 | 1.065 |

Note: Variables with a GVIF greater than 5 or with an adjusted GVIF (GVIF^(1/(2*Df))) greater than 2 were considered collinear and were excluded from the multiple regression model

**Table S3** Multicollinearity analysis in the association analysis between eGDR and CMM

| Variables | GVIF | Df | GVIF^(1/(2*Df)) |
| --- | --- | --- | --- |
| eGDR | 1.691 | 1 | 1.300 |
| Age | 1.412 | 1 | 1.188 |
| Sex | 2.332 | 1 | 1.527 |
| Smoking status | 1.849 | 1 | 1.360 |
| Drinking status | 1.485 | 1 | 1.219 |
| Education level | 1.378 | 3 | 1.055 |
| Marital status | 1.099 | 1 | 1.048 |
| Residence | 1.102 | 1 | 1.050 |
| SBP | 2.516 | 1 | 1.586 |
| DBP | 2.465 | 1 | 1.570 |
| TC | 3.094 | 1 | 1.759 |
| HDL-C | 1.150 | 1 | 1.072 |
| LDL-C | 3.035 | 1 | 1.742 |
| hs-CRP | 1.012 | 1 | 1.006 |
| Hypertension | 2.940 | 1 | 1.715 |
| Dyslipidemia | 1.842 | 1 | 1.357 |
| Depression | 1.101 | 1 | 1.049 |
| Cancer | 1.036 | 1 | 1.018 |
| Antihypertensive medication | 2.076 | 1 | 1.441 |
| Antidiabetic medication | 1.115 | 1 | 1.056 |
| Antidyslipidemic medication | 1.781 | 1 | 1.335 |
| Cardiovascular medications | 1.139 | 1 | 1.067 |

Note: Variables with a GVIF greater than 5 or with an adjusted GVIF (GVIF^(1/(2*Df))) greater than 2 were considered collinear and were excluded from the multiple regression model

**Table S4** Multicollinearity analysis in the association analysis between METS-IR and CMM

| Variables | GVIF | Df | GVIF^(1/(2*Df)) |
| --- | --- | --- | --- |
| METS-IR | 1.600 | 1 | 1.265 |
| Age | 1.411 | 1 | 1.188 |
| Sex | 2.316 | 1 | 1.522 |
| Smoking status | 1.849 | 1 | 1.360 |
| Drinking status | 1.477 | 1 | 1.215 |
| Education level | 1.379 | 3 | 1.055 |
| Marital status | 1.098 | 1 | 1.048 |
| Residence | 1.103 | 1 | 1.050 |
| SBP | 2.568 | 1 | 1.602 |
| DBP | 2.512 | 1 | 1.585 |
| TC | 3.126 | 1 | 1.768 |
| HDL-C | 1.122 | 1 | 1.059 |
| LDL-C | 3.075 | 1 | 1.753 |
| hs-CRP | 1.011 | 1 | 1.005 |
| Hypertension | 2.473 | 1 | 1.573 |
| Dyslipidemia | 1.847 | 1 | 1.359 |
| Depression | 1.097 | 1 | 1.047 |
| Cancer | 1.037 | 1 | 1.018 |
| Antihypertensive medication | 2.082 | 1 | 1.443 |
| Antidiabetic medication | 1.114 | 1 | 1.055 |
| Antidyslipidemic medication | 1.790 | 1 | 1.338 |
| Cardiovascular medications | 1.144 | 1 | 1.069 |

Note: Variables with a GVIF greater than 5 or with an adjusted GVIF (GVIF^(1/(2*Df))) greater than 2 were considered collinear and were excluded from the multiple regression model

**Table S5** Multicollinearity analysis in the association analysis between BMR with TyG and CMM

| Variables | GVIF | Df | GVIF^(1/(2*Df)) |
| --- | --- | --- | --- |
| BMR + TyG | 1.851 | 3 | 1.108 |
| Age | 1.470 | 1 | 1.213 |
| Sex | 2.314 | 1 | 1.521 |
| Smoking status | 1.839 | 1 | 1.356 |
| Drinking status | 1.485 | 1 | 1.218 |
| Education level | 1.379 | 3 | 1.055 |
| Marital status | 1.101 | 1 | 1.049 |
| Residence | 1.111 | 1 | 1.054 |
| SBP | 2.557 | 1 | 1.599 |
| DBP | 2.518 | 1 | 1.587 |
| TC | 3.735 | 1 | 1.933 |
| HDL-C | 1.469 | 1 | 1.212 |
| LDL-C | 3.370 | 1 | 1.836 |
| hs-CRP | 1.013 | 1 | 1.006 |
| Hypertension | 2.453 | 1 | 1.566 |
| Dyslipidemia | 1.850 | 1 | 1.360 |
| Depression | 1.104 | 1 | 1.051 |
| Cancer | 1.036 | 1 | 1.018 |
| Antihypertensive medication | 2.097 | 1 | 1.448 |
| Antidiabetic medication | 1.112 | 1 | 1.054 |
| Antidyslipidemic medication | 1.781 | 1 | 1.335 |
| Cardiovascular medications | 1.136 | 1 | 1.066 |

Note: Variables with a GVIF greater than 5 or with an adjusted GVIF (GVIF^(1/(2*Df))) greater than 2 were considered collinear and were excluded from the multiple regression model

**Table S6** Multicollinearity analysis in the association analysis between BMR with eGDR and CMM

| Variables | GVIF | Df | GVIF^(1/(2*Df)) |
| --- | --- | --- | --- |
| BMR + eGDR | 2.141 | 3 | 1.135 |
| Age | 1.475 | 1 | 1.214 |
| Sex | 2.326 | 1 | 1.525 |
| Smoking status | 1.841 | 1 | 1.357 |
| Drinking status | 1.491 | 1 | 1.221 |
| Education level | 1.381 | 3 | 1.055 |
| Marital status | 1.100 | 1 | 1.049 |
| Residence | 1.110 | 1 | 1.054 |
| SBP | 2.537 | 1 | 1.593 |
| DBP | 2.490 | 1 | 1.578 |
| TC | 3.051 | 1 | 1.747 |
| HDL-C | 1.199 | 1 | 1.095 |
| LDL-C | 3.001 | 1 | 1.732 |
| hs-CRP | 1.013 | 1 | 1.006 |
| Hypertension | 3.011 | 1 | 1.735 |
| Dyslipidemia | 1.849 | 1 | 1.360 |
| Depression | 1.107 | 1 | 1.052 |
| Cancer | 1.034 | 1 | 1.017 |
| Antihypertensive medication | 2.106 | 1 | 1.451 |
| Antidiabetic medication | 1.116 | 1 | 1.056 |
| Antidyslipidemic medication | 1.779 | 1 | 1.334 |
| Cardiovascular medications | 1.139 | 1 | 1.067 |

Note: Variables with a GVIF greater than 5 or with an adjusted GVIF (GVIF^(1/(2*Df))) greater than 2 were considered collinear and were excluded from the multiple regression model

**Table S7** Multicollinearity analysis in the association analysis between BMR with METS-IR and CMM

| Variables | GVIF | Df | GVIF^(1/(2*Df)) |
| --- | --- | --- | --- |
| BMR + METS-IR | 1.808 | 3 | 1.104 |
| Age | 1.139 | 1 | 1.067 |
| Sex | 1.139 | 1 | 1.067 |
| Smoking status | 1.853 | 1 | 1.361 |
| Drinking status | 1.496 | 1 | 1.223 |
| Education level | 1.388 | 3 | 1.056 |
| Marital status | 1.099 | 1 | 1.049 |
| Residence | 1.108 | 1 | 1.053 |
| SBP | 2.521 | 1 | 1.588 |
| DBP | 2.485 | 1 | 1.576 |
| TC | 3.121 | 1 | 1.767 |
| HDL-C | 1.564 | 1 | 1.251 |
| LDL-C | 3.046 | 1 | 1.745 |
| hs-CRP | 1.012 | 1 | 1.006 |
| Hypertension | 2.461 | 1 | 1.569 |
| Dyslipidemia | 1.848 | 1 | 1.359 |
| Depression | 1.105 | 1 | 1.051 |
| Cancer | 1.036 | 1 | 1.018 |
| Antihypertensive medication | 2.098 | 1 | 1.449 |
| Antidiabetic medication | 1.107 | 1 | 1.052 |
| Antidyslipidemic medication | 1.781 | 1 | 1.335 |
| Cardiovascular medications | 1.137 | 1 | 1.066 |

Note: Variables with a GVIF greater than 5 or with an adjusted GVIF (GVIF^(1/(2*Df))) greater than 2 were considered collinear and were excluded from the multiple regression model

**Table S8** Associations of BMR, TyG, eGDR, and METS-IR with CMM

| **Subgroups** | **Incidence rate ^a^** | **Model I** | | **Model II** | | **Model III** | |
| --- | --- | --- | --- | --- | --- | --- | --- |
|  |  | **HR (95% CI)** | ***P* value** | **HR (95% CI)** | ***P* value** | **HR (95% CI)** | ***P* value** |
| **BMR** | | | | | | | |
| Low | 11.57 | Reference | — | Reference | — | Reference | — |
| High | 18.88 | 1.71 (1.51, 1.93) | < 0.001 | 1.93 (1.70, 2.20) | < 0.001 | 1.42 (1.23, 1.63) | < 0.001 |
| Per 1.0 SD |  | 1.00 (1.00, 1.00) | < 0.001 | 1.00 (1.00, 1.00) | < 0.001 | 1.00 (1.00, 1.00) | < 0.001 |
| **TyG** | | | | | | | |
| Low | 10.44 | Reference | — | Reference | — | Reference | — |
| High | 20.05 | 2.03 (1.79, 2.30) | < 0.001 | 2.01 (1.77, 2.28) | < 0.001 | 1.52 (1.30, 1.77) | < 0.001 |
| Per 1.0 SD |  | 1.78 (1.64, 1.93) | < 0.001 | 1.80 (1.66, 1.95) | < 0.001 | 1.71 (1.39, 2.11) | < 0.001 |
| **eGDR** | | | | | | | |
| Low | 22.48 | Reference | — | Reference | — | Reference | — |
| High | 8.00 | 0.33 (0.28, 0.37) | < 0.001 | 0.34 (0.30, 0.39) | < 0.001 | 0.65 (0.54, 0.78) | < 0.001 |
| Per 1.0 SD |  | 0.75 (0.73, 0.77) | < 0.001 | 0.76 (0.74, 0.78) | < 0.001 | 0.83 (0.77, 0.88) | < 0.001 |
| **METS-IR** | | | | | | | |
| Low | 9.58 | Reference | — | Reference | — | Reference | — |
| High | 20.91 | 2.34 (2.06, 2.66) | < 0.001 | 2.46 (2.16, 2.81) | < 0.001 | 1.82 (1.55, 2.15) | < 0.001 |
| Per 1.0 SD |  | 1.00 (1.00, 1.00) | 0.015 | 1.00 (1.00, 1.00) | 0.008 | 1.00 (1.00, 1.00) | 0.382 |

Model I: non-adjusted

Model II: adjusted for age, gender, smoking status, drinking status, education level, marital status, residence

Model III: further adjusted for SBP, DBP, TC, HDL-C, LDL-C, hs-CRP, chronic diseases (hypertension, dyslipidemia, depression, and cancer), and medication use (antihypertensive, antidiabetic, antidyslipidemic, and cardiovascular medications)

Each index was dichotomized at its median value. *IR* insulin resistance, *BMR* basal metabolic rate, *TyG* triglyceride-glucose, *eGDR* estimate glucose disposal rate, *METS-IR* metabolic score for insulin resistance, *HR* hazard ratio, *CI* confidence interval

**^a^** Incident rate was presented as per 1000 person-years of follow-up

**Table S9**  Threshold effect analysis of the METS-IR on the risk of CMM among participants with high BMR

| **Participants with low BMR** | **Adjusted HR (95% CI)** | ***P* value** |
| --- | --- | --- |
| **METS-IR** |  |  |
| Total | 1.00 (0.99, 1.01) | 0.656 |
| Fitting by two-piecewise Cox proportional risk model |  |  |
| Inflection point | 51.90 |  |
| METS-IR < 51.90 | 1.05 (1.03, 1.07) | < 0.001 |
| METS-IR ≥ 51.90 | 0.98 (0.95, 1.02) | 0.306 |
| *P* for log-likelihood ratio |  | < 0.001 |

Cox proportional hazards models were employed to estimate HRs and 95% CIs. The multivariate models were adjusted for age, gender, smoking status, drinking status, education level, marital status, residence, SBP, DBP, TC, HDL-C, LDL-C, hs-CRP, chronic diseases (including hypertension, dyslipidemia, depression, and cancer), as well as medication use (antihypertensive, antidiabetic, antidyslipidemic, and cardiovascular medications). *METS-IR* metabolic score for insulin resistance, *HRs* hazard ratios, *CIs* confidence intervals

**Table S10** Predictive performance of BMR, TyG, eGDR and METS-IR for CMM

|  | AUC (95%CI) | *P* value | NRI (95%CI) | *P* value | IDI (95%CI) | *P* value |
| --- | --- | --- | --- | --- | --- | --- |
| Basic model | 0.741 (0.725-0.757) | Reference | Reference | — | Reference | — |
| Basic model + BMR | 0.747 (0.731-0.763) | 0.003 | 0.177 (0.111-0.242) | < 0.001 | 0.004 (0.002-0.007) | 0.001 |
| Basic model + TyG | 0.754 (0.738-0.770) | < 0.001 | 0.331 (0.266-0.395) | < 0.001 | 0.014 (0.010-0.019) | < 0.001 |
| Basic model + eGDR | 0.753 (0.734-0.769) | 0.002 | 0.342 (0.277-0.406) | < 0.001 | 0.012 (0.008-0.016) | < 0.001 |
| Basic model + METS-IR | 0.741 (0.725-0.758) | 0.842 | -0.049 (-0.109-0.010) | 0.103 | -0.001 (-0.001-0.000) | 0.086 |
| Basic model + BMR + TyG | 0.759 (0.743-0.775) | < 0.001 | 0.371 (0.306-0.436) | < 0.001 | 0.017 (0.012-0.022) | < 0.001 |
| Basic model + BMR + eGDR | 0.753 (0.734-0.769) | 0.001 | 0.330 (0.266-0.395) | < 0.001 | 0.012 (0.008-0.016) | < 0.001 |
| Basic model + BMR + METS-IR | 0.747 (0.731-0.763) | 0.005 | 0.170 (0.105-0.235) | < 0.001 | 0.004 (0.001-0.006) | 0.007 |

*BMR* basal metabolic rate, *TyG* triglyceride-glucose, *eGDR* estimated glucose disposal rate, *METS-IR* metabolic score for insulin resistance, *AUC* area under the receiver operating characteristic curve, *CI* confidence interval

**Table S11** Predictive performance of BMR **_(H-B)_**, TyG, eGDR and METS-IR for CMM

|  | AUC (95%CI) | *P* value | NRI (95%CI) | *P* value | IDI (95%CI) | *P* value |
| --- | --- | --- | --- | --- | --- | --- |
| Basic model | 0.741 (0.725-0.757) | Reference | Reference | **—** | Reference | **—** |
| Basic model + BMR **_(H-B)_** | 0.744 (0.728-0.760) | 0.131 | 0.110 (0.045-0.175) | 0.001 | 0.002 (0.000-0.004) | 0.030 |
| Basic model + TyG | 0.754 (0.738-0.770) | < 0.001 | 0.331 (0.266-0.395) | < 0.001 | 0.014 (0.010-0.019) | < 0.001 |
| Basic model + eGDR | 0.753 (0.734-0.769) | 0.002 | 0.342 (0.277-0.406) | < 0.001 | 0.012 (0.008-0.016) | < 0.001 |
| Basic model + METS-IR | 0.741 (0.725-0.758) | 0.842 | -0.049 (-0.109-0.010) | 0.103 | -0.001 (-0.001-0.000) | 0.086 |
| Basic model + BMR **_(H-B)_** + TyG | 0.756 (0.740-0.772) | < 0.001 | 0.361 (0.296-0.426) | < 0.001 | 0.016 (0.011-0.021) | < 0.001 |
| Basic model + BMR **_(H-B_**_)_ + eGDR | 0.753 (0.737-0.769) | 0.002 | 0.343 (0.278-0.408) | < 0.001 | 0.012 (0.008-0.016) | < 0.001 |
| Basic model + BMR **_(H-B)_** + METS-IR | 0.744 (0.728-0.760) | 0.104 | 0.116 (0.051-0.181) | 0.001 | 0.002 (0.000-0.004) | 0.036 |

*BMR* **_(H-B)_** basal metabolic rate estimated via Harris-Benedict equation, *TyG* triglyceride-glucose, *eGDR* estimated glucose disposal rate, *METS-IR* metabolic score for insulin resistance, *AUC* area under the receiver operating characteristic curve, *CI* confidence interval

**Table S12** Predictive performance of BMR **_(M-SJ)_**, TyG, eGDR and METS-IR for CMM

|  | AUC (95%CI) | *P* value | NRI (95%CI) | *P* value | IDI (95%CI) | *P* value |
| --- | --- | --- | --- | --- | --- | --- |
| Basic model | 0.741 (0.725-0.757) | Reference | Reference | **—** | Reference | **—** |
| Basic model + BMR **_(M-SJ)_** | 0.749 (0.733-0.765) | 0.003 | 0.166 (0.101-0.231) | < 0.001 | 0.004 (0.001-0.008) | 0.007 |
| Basic model + TyG | 0.754 (0.738-0.770) | < 0.001 | 0.331 (0.266-0.395) | < 0.001 | 0.014 (0.010-0.019) | < 0.001 |
| Basic model + eGDR | 0.753 (0.734-0.769) | 0.002 | 0.342 (0.277-0.406) | < 0.001 | 0.012 (0.008-0.016) | < 0.001 |
| Basic model + METS-IR | 0.741 (0.725-0.758) | 0.842 | -0.049 (-0.109-0.010) | 0.103 | -0.001 (-0.001-0.000) | 0.086 |
| Basic model + BMR **_(M-SJ)_** + TyG | 0.761 (0.745-0.776) | < 0.001 | 0.335 (0.271-0.400) | < 0.001 | 0.017 (0.012-0.023) | < 0.001 |
| Basic model + BMR **_(M-SJ_**_)_ + eGDR | 0.755 (0.740-0.771) | < 0.001 | 0.310 (0.246-0.375) | < 0.001 | 0.013 (0.009-0.018) | < 0.001 |
| Basic model + BMR **_(M-SJ)_** + METS-IR | 0.749 (0.733-0.765) | 0.003 | 0.177 (0.112-0.242) | < 0.001 | 0.004 (0.001-0.008) | 0.007 |

*BMR* **_(M-SJ)_** basal metabolic rate estimated via Mifflin-St Jeor equation, *TyG* triglyceride-glucose, *eGDR* estimated glucose disposal rate, *METS-IR* metabolic score for insulin resistance, *AUC* area under the receiver operating characteristic curve, *CI* confidence interval

**Table S13** Interaction of insulin resistance surrogate indices with basal metabolic rate on CMM risk

| **Interactive indices** | **Interactive effects (95% CI)** | | |
| --- | --- | --- | --- |
|  | **Model I** | **Model II** | **Model III** |
| **BMR & TyG** | | | |
| Additive effect | | | |
| RERI | 0.37 (-0.07, 0.80) | 0.49 (0.02, 0.96) | 0.02 (-0.38, 0.41) |
| AP | 0.13 (-0.02, 0.28) | 0.15 (0.01, 0.30) | 0.01 (-0.18, 0.19) |
| SI | 1.24 (0.93, 1.66) | 1.29 (0.98, 1.69) | 1.01 (0.71, 1.45) |
| Multiplicative effect | 0.95 (0.73, 1.23) | 0.94 (0.72, 1.21) | 0.88 (0.68, 1.14) |
| **BMR & eGDR** | | | |
| Additive effect | | | |
| RERI | 0.27 (-0.27, 0.81) | 0.56 (0.03, 1.09) | 0.29 (-0.09, 0.67) |
| AP | 0.08 (-0.08, 0.25) | 0.17 (0.01, 0.32) | 0.15 (-0.05, 0.35) |
| SI | 1.14 (0.87, 1.49) | 1.31 (0.98, 1.75) | 1.45 (0.79, 2.69) |
| Multiplicative effect | 1.03 (0.77, 1.39) | 1.06 (0.78, 1.43) | 1.12 (0.83, 1.52) |
| **BMR & METS-IR** | | | |
| Additive effect | | | |
| RERI | 0.12 (-0.38, 0.61) | 0.22 (-0.31, 0.75) | -0.12 (-0.59, 0.35) |
| AP | 0.05 (-0.16, 0.26) | 0.08 (-0.11, 0.28) | -0.06 (-0.31, 0.18) |
| SI | 1.09 (0.74, 1.61) | 1.15 (0.80, 1.65) | 0.88 (0.56, 1.39) |
| Multiplicative effect | 1.03 (0.74, 1.42) | 0.97 (0.70, 1.34) | 0.86 (0.63, 1.19) |

Model I: non-adjusted

Model II: adjusted for age, gender, smoking status, drinking status, education level, marital status, residence

Model III: further adjusted for SBP, DBP, TC, HDL-C, LDL-C, hs-CRP, chronic diseases (hypertension, dyslipidemia, depression, and cancer), and medication use (antihypertensive, antidiabetic, antidyslipidemic, and cardiovascular medications)

*BMR* basal metabolic rate, *TyG* triglyceride-glucose, *eGDR* estimate glucose disposal rate, *METS-IR* metabolic score for insulin resistance, *RERI* relative excess risk due to interaction, *AP* attributable proportion due to interaction, *SI* synergy index

**Table S14** Sensitivity analyses of the joint effects of insulin resistance surrogate indices and basal metabolic rate on the risks of developing CMM (*N*=6974, BMR estimated via Harris-Benedict equation)

| **Subgroups** | **Incidence rate ^a^** | **Model I** | | **Model II** | | **Model III** | |
| --- | --- | --- | --- | --- | --- | --- | --- |
|  |  | **HR (95% CI)** | ***P* value** | **HR (95% CI)** | ***P* value** | **HR (95% CI)** | ***P* value** |
| **TyG and BMR** | | | | | | | |
| Group 1 | 10.42 | Reference | — | Reference | — | Reference | — |
| Group 2 | 10.46 | 1.01 (0.82, 1.24) | 0.945 | 1.37 (1.10, 1.70) | 0.005 | 1.18 (0.94, 1.47) | 0.146 |
| Group 3 | 18.48 | 1.86 (1.55, 2.22) | < 0.001 | 1.78 (1.48, 2.13) | < 0.001 | 1.50 (1.23, 1.83) | < 0.001 |
| Group 4 | 21.19 | 2.17 (1.84, 2.56) | < 0.001 | 2.76 (2.32, 3.29) | < 0.001 | 1.78 (1.45, 2.19) | < 0.001 |
| *P* for trend |  |  | < 0.001 |  | < 0.001 |  | < 0.001 |
| **eGDR and BMR** | | | | | | | |
| Group 1 | 8.62 | Reference | — | Reference | — | Reference | — |
| Group 2 | 6.85 | 0.79 (0.61, 1.02) | 0.071 | 1.01 (0.77, 1.32) | 0.943 | 0.96 (0.74, 1.25) | 0.771 |
| Group 3 | 23.60 | 3.01 (2.51, 3.61) | < 0.001 | 2.61 (2.17, 3.14) | < 0.001 | 1.56 (1.25, 1.94) | < 0.001 |
| Group 4 | 21.89 | 2.76 (2.34, 3.26) | < 0.001 | 3.13 (2.64, 3.71) | < 0.001 | 1.78 (1.45, 2.19) | < 0.001 |
| *P* for trend |  |  | < 0.001 |  | < 0.001 |  | < 0.001 |
| **METS-IR and BMR** | | | | | | | |
| Group 1 | 10.46 | Reference | — | Reference | — | Reference | — |
| Group 2 | 7.07 | 0.66 (0.51, 0.87) | 0.003 | 0.93 (0.70, 1.24) | 0.628 | 0.97 (0.73, 1.29) | 0.848 |
| Group 3 | 23.43 | 2.42 (2.02, 2.90) | < 0.001 | 2.24 (1.87, 2.69) | < 0.001 | 1.87 (1.52, 2.30) | < 0.001 |
| Group 4 | 20.02 | 2.03 (1.76, 2.36) | < 0.001 | 2.53 (2.16, 2.96) | < 0.001 | 1.78 (1.49, 2.14) | < 0.001 |
| *P* for trend |  |  | < 0.001 |  | < 0.001 |  | < 0.001 |

Model I: non-adjusted

Model II: adjusted for age, gender, smoking status, drinking status, education level, marital status, residence

Model III: further adjusted for SBP, DBP, TC, HDL-C, LDL-C, hs-CRP, chronic diseases (hypertension, dyslipidemia, depression, and cancer), and medication use (antihypertensive, antidiabetic, antidyslipidemic, and cardiovascular medications)

Group 1 refers to low IR and low BMR; Group 2 refers to low IR and high BMR; Group 3 refers to high IR and low BMR; Group 4 refers to high IR and high BMR. *IR* insulin resistance, *BMR* basal metabolic rate, *TyG* triglyceride-glucose, *eGDR* estimate glucose disposal rate, *METS-IR* metabolic score for insulin resistance, *HR* hazard ratio, *CI* confidence interval

**^a^** Incident rate was presented as per 1000 person-years of follow-up

**Table S15** Sensitivity analyses of the joint effects of insulin resistance surrogate indices and basal metabolic rate on the risks of developing CMM (*N*=6974, BMR estimated via Mifflin-St. Jeor equation)

| **Subgroups** | **Incidence rate ^a^** | **Model I** | | **Model II** | | **Model III** | |
| --- | --- | --- | --- | --- | --- | --- | --- |
|  |  | **HR (95% CI)** | ***P* value** | **HR (95% CI)** | ***P* value** | **HR (95% CI)** | ***P* value** |
| **TyG and BMR** | | | | | | | |
| Group 1 | 10.34 | Reference | — | Reference | — | Reference | — |
| Group 2 | 10.57 | 1.03 (0.83, 1.26) | 0.804 | 1.39 (1.12, 1.73) | 0.003 | 1.22 (0.98, 1.52) | 0.078 |
| Group 3 | 18.06 | 1.83 (1.52, 2.19) | < 0.001 | 1.76 (1.47, 2.11) | < 0.001 | 1.49 (1.22, 1.82) | < 0.001 |
| Group 4 | 21.54 | 2.23 (1.89, 2.63) | < 0.001 | 2.84 (2.39, 3.38) | < 0.001 | 1.85 (1.51, 2.28) | < 0.001 |
| *P* for trend |  |  | < 0.001 |  | < 0.001 |  | < 0.001 |
| **eGDR and BMR** | | | | | | | |
| Group 1 | 8.67 | Reference | — | Reference | — | Reference | — |
| Group 2 | 6.80 | 0.78 (0.60, 1.00) | 0.053 | 1.00 (0.77, 1.30) | 0.976 | 0.95 (0.73, 1.23) | 0.685 |
| Group 3 | 22.56 | 2.84 (2.36, 3.41) | < 0.001 | 2.47 (2.06, 2.98) | < 0.001 | 1.48 (1.19, 1.85) | < 0.001 |
| Group 4 | 22.44 | 2.82 (2.39, 3.33) | < 0.001 | 3.22 (2.71, 3.82) | < 0.001 | 1.81 (1.48, 2.23) | < 0.001 |
| *P* for trend |  |  | < 0.001 |  | < 0.001 |  | < 0.001 |
| **METS-IR and BMR** | | | | | | | |
| Group 1 | 10.46 | Reference | — | Reference | — | Reference | — |
| Group 2 | 7.30 | 0.69 (0.53, 0.89) | 0.005 | 0.96 (0.73, 1.26) | 0.783 | 1.01 (0.77, 1.32) | 0.942 |
| Group 3 | 21.91 | 2.24 (1.87, 2.69) | < 0.001 | 2.12 (1.77, 2.55) | < 0.001 | 1.79 (1.46, 2.19) | < 0.001 |
| Group 4 | 20.52 | 2.09 (1.80, 2.42) | < 0.001 | 2.63 (2.25, 3.08) | < 0.001 | 1.85 (1.54, 2.22) | < 0.001 |
| *P* for trend |  |  | < 0.001 |  | < 0.001 |  | < 0.001 |

Model I: non-adjusted

Model II: adjusted for age, gender, smoking status, drinking status, education level, marital status, residence

Model III: further adjusted for SBP, DBP, TC, HDL-C, LDL-C, hs-CRP, chronic diseases (hypertension, dyslipidemia, depression, and cancer), and medication use (antihypertensive, antidiabetic, antidyslipidemic, and cardiovascular medications)

Group 1 refers to low IR and low BMR; Group 2 refers to low IR and high BMR; Group 3 refers to high IR and low BMR; Group 4 refers to high IR and high BMR. *IR* insulin resistance, *BMR* basal metabolic rate, *TyG* triglyceride-glucose, *eGDR* estimate glucose disposal rate, *METS-IR* metabolic score for insulin resistance, *HR* hazard ratio, *CI* confidence interval

**^a^** Incident rate was presented as per 1000 person-years of follow-up

**Table S16** Sensitivity analyses of the joint effects of insulin resistance surrogate indices and basal metabolic rate on the risks of developing CMM in participants who were free of the outcome within the first 2 years (*N*=6,870).

| **Subgroups** | **Incidence rate ^a^** | **Model I** | | **Model II** | | **Model III** | |
| --- | --- | --- | --- | --- | --- | --- | --- |
|  |  | **HR (95% CI)** | ***P* value** | **HR (95% CI)** | ***P* value** | **HR (95% CI)** | ***P* value** |
| **TyG and BMR** | | | | | | | |
| Group 1 | 7.94 | Reference | — | Reference | — | Reference | — |
| Group 2 | 11.96 | 1.54 (1.24, 1.91) | < 0.001 | 1.78 (1.43, 2.22) | < 0.001 | 1.48 (1.18, 1.85) | 0.001 |
| Group 3 | 14.90 | 1.95 (1.59,2.39) | < 0.001 | 1.90 (1.54, 2.33) | < 0.001 | 1.60 (1.28, 2.00) | < 0.001 |
| Group 4 | 20.75 | 2.81 (2.35, 3.37) | < 0.001 | 3.14 (2.61, 3.78) | < 0.001 | 2.09 (1.68, 2.61) | < 0.001 |
| *P* for trend |  |  | < 0.001 |  | < 0.001 |  | < 0.001 |
| **eGDR and BMR** | | | | | | | |
| Group 1 | 7.36 | Reference | — | Reference | — | Reference | — |
| Group 2 | 7.65 | 1.04 (0.80, 1.36) | 0.780 | 1.20 (0.92, 1.57) | 0.186 | 1.12 (0.85, 1.47) | 0.411 |
| Group 3 | 19.18 | 2.80 (2.28, 3.44) | < 0.001 | 2.46 (2.00, 3.02) | < 0.001 | 1.40 (1.09, 1.80) | 0.008 |
| Group 4 | 21.16 | 3.13 (2.64, 3.70) | < 0.001 | 3.26 (2.74, 3.87) | < 0.001 | 1.85 (1.50, 2.28) | < 0.001 |
| *P* for trend |  |  | < 0.001 |  | < 0.001 |  | < 0.001 |
| **METS-IR and BMR** | | | | | | | |
| Group 1 | 8.84 | Reference | — | Reference | — | Reference | — |
| Group 2 | 9.26 | 1.05 (0.80, 1.38) | 0.739 | 1.26 (0.96, 1.66) | 0.102 | 1.24 (0.94, 1.63) | 0.131 |
| Group 3 | 18.40 | 2.20 (1.77, 2.73) | < 0.001 | 2.19 (1.76, 2.72) | < 0.001 | 1.75 (1.38, 2.23) | < 0.001 |
| Group 4 | 19.24 | 2.32 (1.99, 2.70) | < 0.001 | 2.62 (2.24, 3.06) | < 0.001 | 1.84 (1.53, 2.22) | < 0.001 |
| *P* for trend |  |  | < 0.001 |  | < 0.001 |  | < 0.001 |

Model I: non-adjusted

Model II: adjusted for age, gender, smoking status, drinking status, education level, marital status, residence

Model III: further adjusted for SBP, DBP, TC, HDL-C, LDL-C, hs-CRP, chronic diseases (hypertension, dyslipidemia, depression, and cancer), and medication use (antihypertensive, antidiabetic, antidyslipidemic, and cardiovascular medications)

Group 1 refers to low IR and low BMR; Group 2 refers to low IR and high BMR; Group 3 refers to high IR and low BMR; Group 4 refers to high IR and high BMR. *IR* insulin resistance, *BMR* basal metabolic rate, *TyG* triglyceride-glucose, *eGDR* estimate glucose disposal rate, *METS-IR* metabolic score for insulin resistance, *HR* hazard ratio, *CI* confidence interval

**^a^** Incident rate was presented as per 1000 person-years of follow-up

**Table S17** Sensitivity analyses **regarding** the joint effects of insulin resistance surrogate indices and basal metabolic rate on the risks of developing CMM **with respect to missing data**.

| **Subgroups** | **Incidence rate ^a^** | **Sensitivity analysis 1** | | **Incidence rate ^a^** | **Sensitivity analysis 2** | |
| --- | --- | --- | --- | --- | --- | --- |
|  |  | **HR (95% CI)** | ***P* value** |  | **HR (95% CI)** | ***P* value** |
| **TyG and BMR** |  | | | | | |
| Group 1 | 8.61 | Reference | — | 8.72 | Reference | — |
| Group 2 | 13.15 | 1.51 (1.22, 1.87) | < 0.001 | 13.03 | 1.48 (1.20, 1.83) | < 0.001 |
| Group 3 | 16.01 | 1.61 (1.30, 1.99) | < 0.001 | 16.38 | 1.62 (1.32, 1.99) | < 0.001 |
| Group 4 | 22.71 | 2.13 (1.73, 2.63) | < 0.001 | 22.66 | 2.11 (1.72, 2.58) | < 0.001 |
| *P* for trend |  |  | < 0.001 |  |  | < 0.001 |
| **eGDR and BMR** | |  | | | | |
| Group 1 | 7.81 | Reference | — | 8.04 | Reference | — |
| Group 2 | 8.46 | 1.17 (0.90, 1.51) | 0.236 | 8.37 | 1.12 (0.87, 1.45) | 0.369 |
| Group 3 | 20.88 | 1.47 (1.16, 1.86) | 0.002 | 21.11 | 1.43 (1.13, 1.81) | 0.002 |
| Group 4 | 23.12 | 1.93 (1.57, 2.36) | 0.001 | 23.04 | 1.87 (1.54, 2.28) | < 0.001 |
| *P* for trend |  |  | < 0.001 |  |  | < 0.001 |
| **METS-IR and BMR** | | | | | | |
| Group 1 | 9.50 | Reference | — | 9.74 | Reference | — |
| Group 2 | 9.90 | 1.22 (0.94, 1.60) | 0.140 | 10.04 | 1.21 (0.93, 1.57) | 0.152 |
| Group 3 | 19.94 | 1.81 (1.44, 2.28) | < 0.001 | 20.03 | 1.76 (1.40, 2.20) | < 0.001 |
| Group 4 | 21.14 | 1.92 (1.61, 2.29) | < 0.001 | 21.02 | 1.85 (1.56, 2.20) | < 0.001 |
| *P* for trend |  |  | < 0.001 |  |  | < 0.001 |

Analyses were adjusted for age, gender, smoking status, drinking status, education level, marital status, residence, SBP, DBP, TC, HDL-C, LDL-C, hs-CRP, chronic diseases (including hypertension, dyslipidemia, depression, and cancer), as well as medication use (antihypertensive, antidiabetic, antidyslipidemic, and cardiovascular medications)

Group 1 refers to low IR and low BMR; Group 2 refers to low IR and high BMR; Group 3 refers to high IR and low BMR; Group 4 refers to high IR and high BMR. *IR* insulin resistance, *BMR* basal metabolic rate, *TyG* triglyceride-glucose, *eGDR* estimate glucose disposal rate, *METS-IR* metabolic score for insulin resistance, *HR* hazard ratio, *CI* confidence interval

**^a^** Incident rate was presented as per 1000 person-years of follow-up

Sensitivity analysis 1 was performed among 6,974 participants without any missing data; sensitivity analysis 2 was performed using multiple imputed analyses (5 iterations) by Markov chain Monte Carlo method

**Table S18** Sensitivity analyses of the joint effects of insulin resistance surrogate indices and basal metabolic rate on the risks of developing CMM in participants who were not receiving treatment for diabetes, hypertension, dyslipidemia, heart disease, or stroke at baseline (*N*=5,439)

| **Subgroups** | **Incidence rate ^a^** | **Model I** | | **Model II** | | **Model III** | |
| --- | --- | --- | --- | --- | --- | --- | --- |
|  |  | **HR (95% CI)** | ***P* value** | **HR (95% CI)** | ***P* value** | **HR (95% CI)** | ***P* value** |
| **TyG and BMR** | | | | | | | |
| Group 1 | 6.63 | Reference | — | Reference | — | Reference | — |
| Group 2 | 9.61 | 1.47 (1.14, 1.91) | 0.004 | 1.73 (1.33, 2.26) | < 0.001 | 1.62 (1.23, 2.12) | < 0.001 |
| Group 3 | 12.73 | 1.98 (1.56, 2.53) | < 0.001 | 1.97 (1.54, 2.51) | < 0.001 | 1.70 (1.30, 2.22) | < 0.001 |
| Group 4 | 15.02 | 2.37 (1.90, 2.97) | < 0.001 | 2.74 (2.18, 3.45) | < 0.001 | 2.22 (1.69, 2.90) | < 0.001 |
| *P* for trend |  |  | < 0.001 |  | < 0.001 |  | < 0.001 |
| **eGDR and BMR** | | | | | | | |
| Group 1 | 7.17 | Reference | — | Reference | — | Reference | — |
| Group 2 | 7.43 | 1.04 (0.79, 1.37) | 0.802 | 1.21 (0.91, 1.61) | 0.180 | 1.19 (0.89, 1.58) | 0.236 |
| Group 3 | 16.30 | 2.40 (1.86, 3.08) | < 0.001 | 2.16 (1.68, 2.78) | < 0.001 | 1.50 (1.11, 2.02) | 0.008 |
| Group 4 | 15.80 | 2.33 (1.91, 2.83) | < 0.001 | 2.54 (2.08, 3.10) | < 0.001 | 1.93 (1.53, 2.43) | < 0.001 |
| *P* for trend |  |  | < 0.001 |  | < 0.001 |  | < 0.001 |
| **METS-IR and BMR** | | | | | | | |
| Group 1 | 7.79 | Reference | — | Reference | — | Reference | — |
| Group 2 | 7.67 | 0.98 (0.71, 1.36) | 0.921 | 1.17 (0.84, 1.62) | 0.349 | 1.20 (0.86, 1.66) | 0.277 |
| Group 3 | 14.45 | 1.93 (1.48, 2.52) | < 0.001 | 1.99 (1.53, 2.61) | < 0.001 | 1.67 (1.23, 2.26) | 0.001 |
| Group 4 | 14.17 | 1.89 (1.57, 2.28) | < 0.001 | 2.22 (1.83, 2.69) | < 0.001 | 1.89 (1.51, 2.36) | < 0.001 |
| *P* for trend |  |  | < 0.001 |  | < 0.001 |  | < 0.001 |

Model I: non-adjusted

Model II: adjusted for age, gender, smoking status, drinking status, education level, marital status, residence

Model III: further adjusted for SBP, DBP, TC, HDL-C, LDL-C, hs-CRP, chronic diseases (hypertension, dyslipidemia, depression, and cancer)

Group 1 refers to low IR and low BMR; Group 2 refers to low IR and high BMR; Group 3 refers to high IR and low BMR; Group 4 refers to high IR and high BMR. *IR* insulin resistance, *BMR* basal metabolic rate, *TyG* triglyceride-glucose, *eGDR* estimate glucose disposal rate, *METS-IR* metabolic score for insulin resistance, *HR* hazard ratio, *CI* confidence interval

**^a^** Incident rate was presented as per 1000 person-years of follow-up

**Table S19** Sensitivity analysis of the joint effects of insulin resistance surrogate indices and basal metabolic rate on the risks of developing CMM in participants without diabetes, heart disease or stroke at baseline (*N*=5,237)

| **Subgroups** | **Incidence rate ^a^** | **Model I** | | **Model II** | | **Model III** | |
| --- | --- | --- | --- | --- | --- | --- | --- |
|  |  | **HR (95% CI)** | ***P* value** | **HR (95% CI)** | ***P* value** | **HR (95% CI)** | ***P* value** |
| **TyG and BMR** | | | | | | | |
| Group 1 | 5.35 | Reference | — | Reference | — | Reference | — |
| Group 2 | 8.14 | 1.54 (1.14, 2.08) | 0.005 | 1.80 (1.32, 2.44) | < 0.001 | 1.58 (1.16, 2.16) | 0.004 |
| Group 3 | 8.41 | 1.59 (1.18, 2.14) | 0.002 | 1.57 (1.17, 2.12) | 0.003 | 1.32 (0.95, 1.82) | 0.098 |
| Group 4 | 11.63 | 2.24 (1.73, 2.91) | < 0.001 | 2.63 (2.01, 3.43) | < 0.001 | 1.87 (1.36, 2.57) | < 0.001 |
| *P* for trend |  |  | < 0.001 |  | < 0.001 |  | < 0.001 |
| **eGDR and BMR** | | | | | | | |
| Group 1 | 5.37 | Reference | — | Reference | — | Reference | — |
| Group 2 | 5.15 | 0.96 (0.65, 1.40) | 0.813 | 1.13 (0.77, 1.65) | 0.546 | 1.06 (0.72, 1.56) | 0.768 |
| Group 3 | 9.98 | 1.91 (1.41, 2.59) | < 0.001 | 1.66 (1.22, 2.26) | 0.001 | 1.06 (0.74, 1.52) | 0.744 |
| Group 4 | 12.04 | 2.33 (1.85, 2.94) | < 0.001 | 2.53 (2.00, 3.20) | < 0.001 | 1.70 (1.28, 2.25) | < 0.001 |
| *P* for trend |  |  | < 0.001 |  | < 0.001 |  | < 0.001 |
| **METS-IR and BMR** | | | | | | | |
| Group 1 | 6.12 | Reference | — | Reference | — | Reference | — |
| Group 2 | 5.87 | 0.95 (0.64, 1.41) | 0.806 | 1.14 (0.77, 1.70) | 0.505 | 1.13 (0.76, 1.68) | 0.553 |
| Group 3 | 8.49 | 1.40 (1.00, 1.97) | 0.051 | 1.45 (1.03, 2.05) | 0.031 | 1.20 (0.82, 1.74) | 0.347 |
| Group 4 | 11.30 | 1.90 (1.53, 2.35) | < 0.001 | 2.24 (1.80, 2.80) | < 0.001 | 1.73 (1.33, 2.25) | < 0.001 |
| *P* for trend |  |  | < 0.001 |  | < 0.001 |  | < 0.001 |

Model I: non-adjusted

Model II: adjusted for age, gender, smoking status, drinking status, education level, marital status, residence

Model III: further adjusted for SBP, DBP, TC, HDL-C, LDL-C, hs-CRP, chronic diseases (hypertension, dyslipidemia, depression, and cancer), and medication use (antihypertensive and antidyslipidemic)

Group 1 refers to low IR and low BMR; Group 2 refers to low IR and high BMR; Group 3 refers to high IR and low BMR; Group 4 refers to high IR and high BMR. *IR* insulin resistance, *BMR* basal metabolic rate, *TyG* triglyceride-glucose, *eGDR* estimate glucose disposal rate, *METS-IR* metabolic score for insulin resistance, *HR* hazard ratio, *CI* confidence interval

**^a^** Incident rate was presented as per 1000 person-years of follow-up

**Table S20**  Threshold effect analysis of the BMR on the risk of CMM among female

| **Participants with low BMR** | **Adjusted HR (95% CI)** | ***P* value** |
| --- | --- | --- |
| **BMR** |  |  |
| Total | 1.001 (1.000, 1.002) | < 0.001 |
| Fitting by two-piecewise Cox proportional risk model |  |  |
| Inflection point | 1480.47 |  |
| BMR < 1480.47 | 1.002 (1.001, 1.003) | < 0.001 |
| BMR ≥ 1480.47 | 0.979 (0.963, 0.996) | 0.017 |
| *P* for log-likelihood ratio |  | 0.001 |

Cox proportional hazards models were employed to estimate HRs and 95% CIs. The multivariate models were adjusted for age, smoking status, drinking status, education level, marital status, residence, SBP, DBP, TC, HDL-C, LDL-C, hs-CRP, chronic diseases (including hypertension, dyslipidemia, depression, and cancer), as well as medication use (antihypertensive, antidiabetic, antidyslipidemic, and cardiovascular medications). *BMR* basal metabolic rate, *HRs* hazard ratios, *CIs* confidence intervals


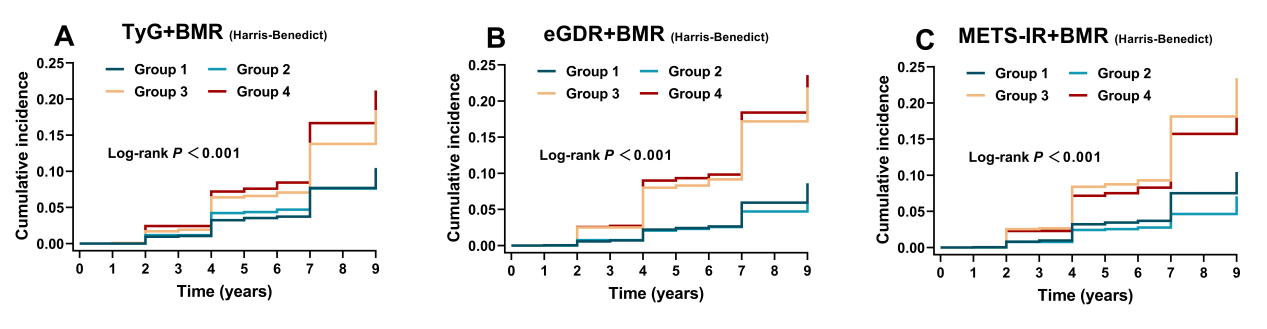


**Fig. S1** Kaplan–Meier curves for the cumulative incidence of CMM, stratified by levels of IR surrogate indices and BMR (A-C). Group 1 refers to low IR and low BMR; Group 2 refers to low IR and high BMR; Group 3 refers to high IR and low BMR; Group 4 refers to high IR and high BMR. *IR* insulin resistance, *BMR* basal metabolic rate, *TyG* triglyceride-glucose, *eGDR* estimate glucose disposal rate, *METS-IR* metabolic score for insulin resistance


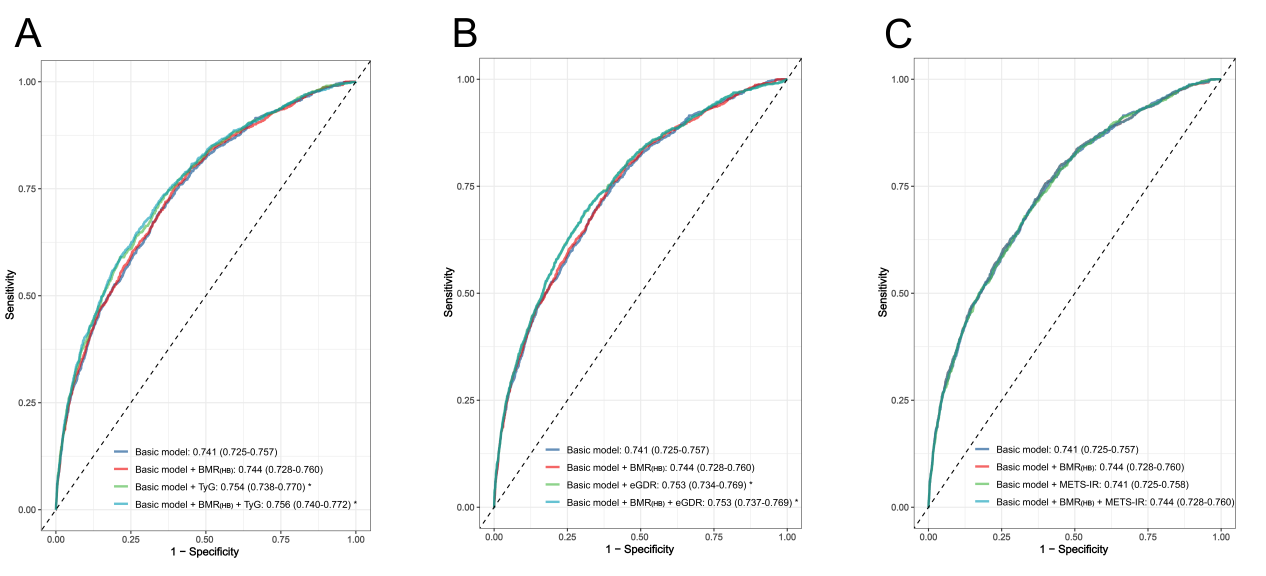


**Fig. S2** ROC curves for CMM prediction, stratified by BMR assessment method. The curves evaluate insulin resistance surrogates (TyG, eGDR, METS-IR) alone and combined with BMR. Panels A-C show the corresponding results for the Harris-Benedict equation, respectively. The basic model was adjusted for age, gender, smoking status, drinking status, education level, marital status, residence, SBP, DBP, TC, HDL-C, LDL-C, hs-CRP, chronic diseases (hypertension, dyslipidemia, depression, and cancer), and medication use (antihypertensive, antidiabetic, antidyslipidemic, and cardiovascular medications). * indicates significantly improved predictive performance versus the basic model by DeLong's test (*P* **<** 0.05)


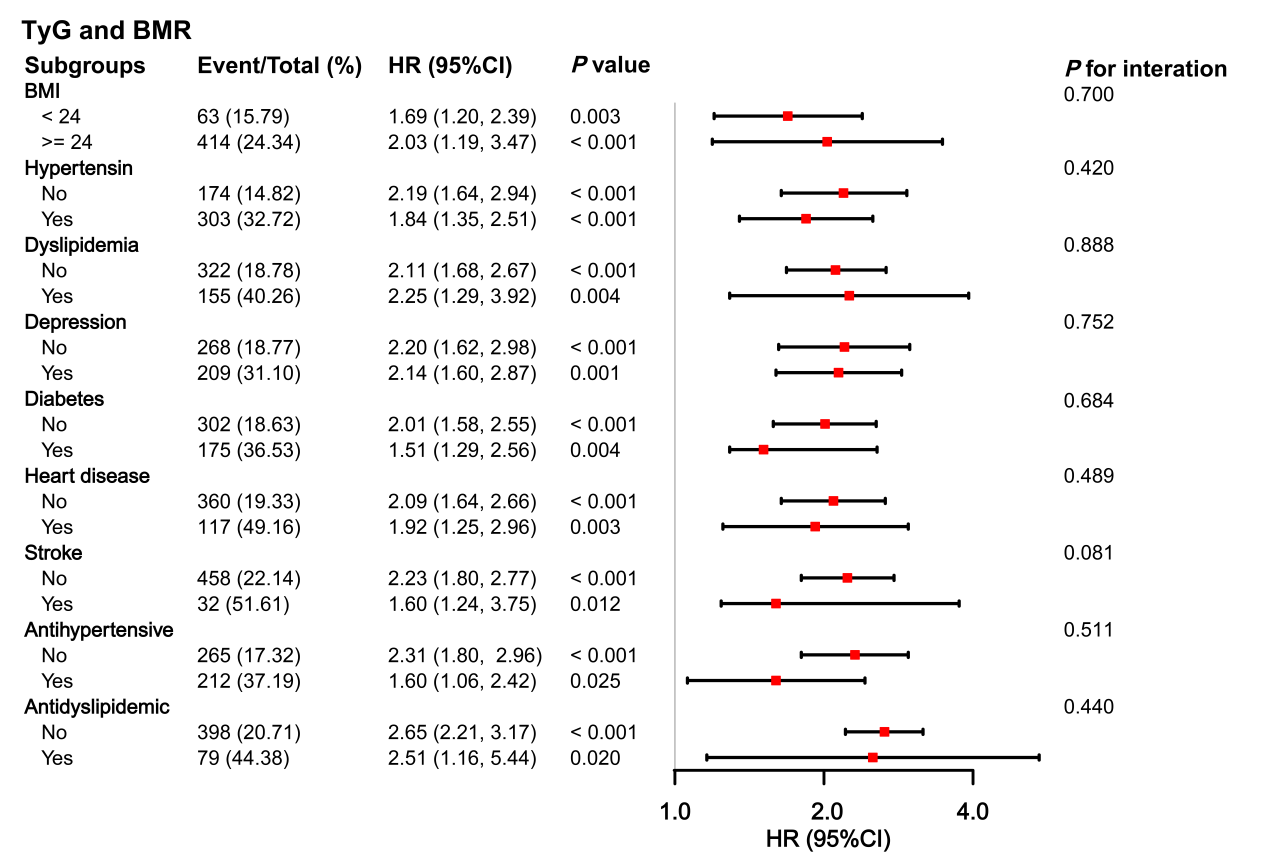


**Fig. S3** Forest plot of multivariable-adjusted Cox regression analyses in the subgroup of participants with both high TyG and high BMR. Multivariate models were adjusted for age, gender, smoking status, drinking status, education level, marital status, residence, SBP, DBP, TC, HDL-C, LDL-C, hs-CRP, chronic diseases (hypertension, dyslipidemia, depression, and cancer), and medication use (antihypertensive, antidiabetic, antidyslipidemic, and cardiovascular medications), with the exception of the stratification variable.


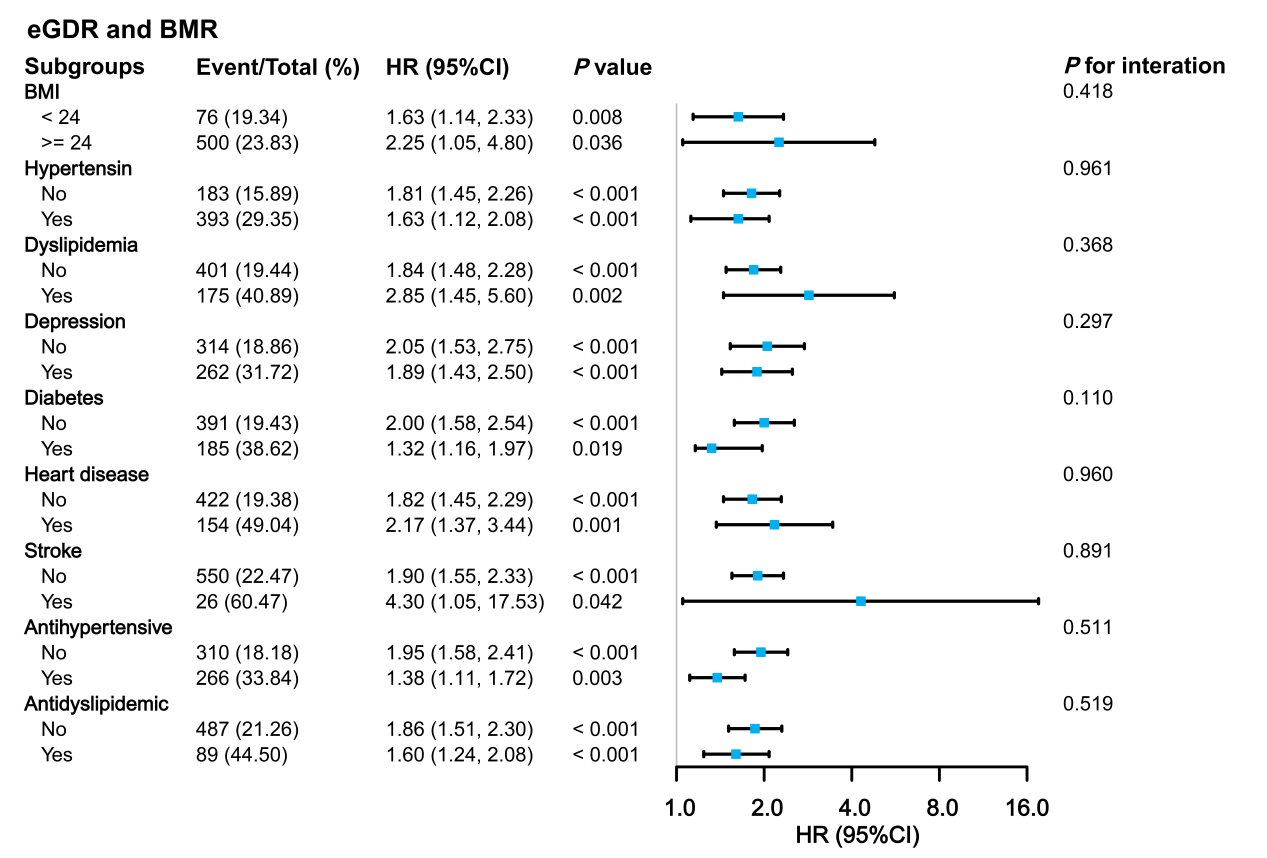


**Fig. S4** Forest plot of multivariable-adjusted Cox regression analyses in the subgroup of participants with both high eGDR and high BMR. Multivariate models were adjusted for age, gender, smoking status, drinking status, education level, marital status, residence, SBP, DBP, TC, HDL-C, LDL-C, hs-CRP, chronic diseases (hypertension, dyslipidemia, depression, and cancer), and medication use (antihypertensive, antidiabetic, antidyslipidemic, and cardiovascular medications), with the exception of the stratification variable.


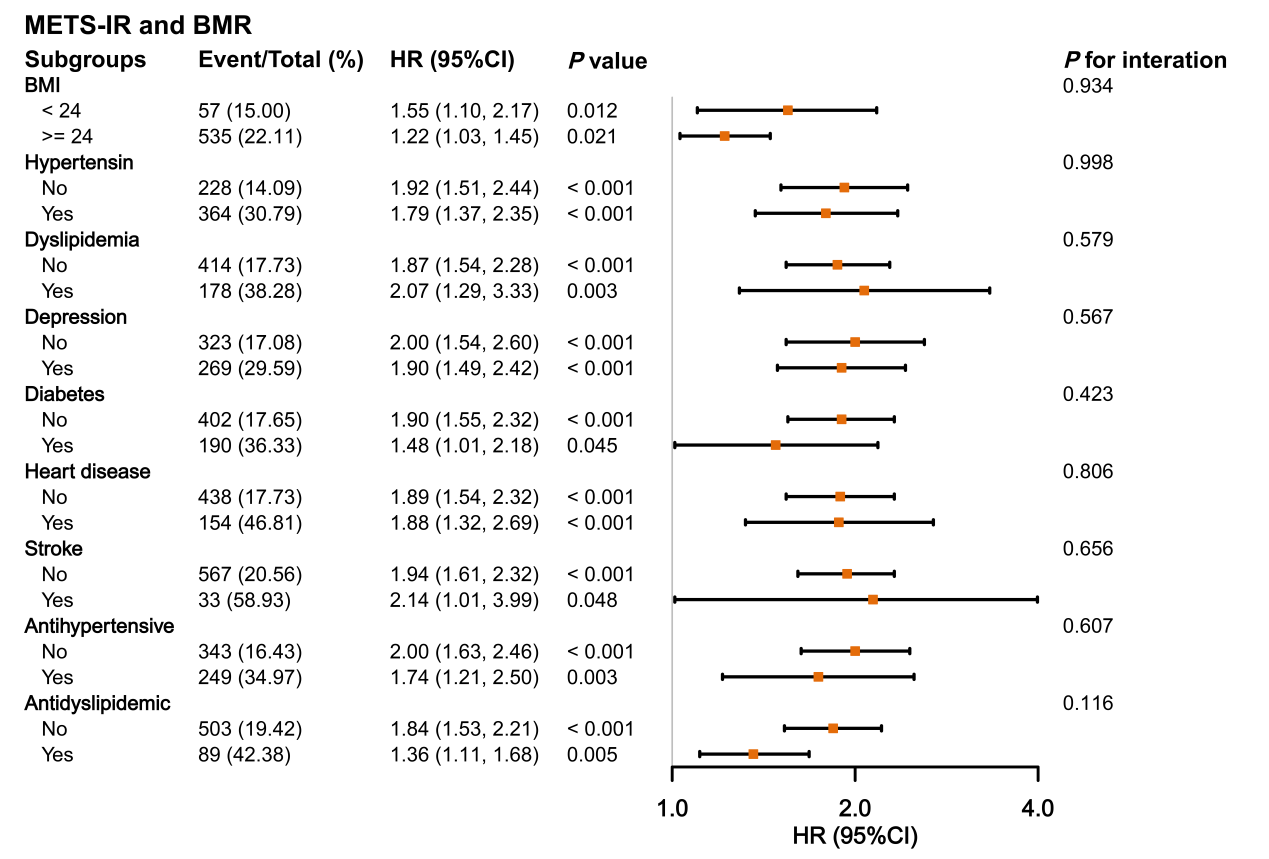


**Fig. S5** Forest plot of multivariable-adjusted Cox regression analyses in the subgroup of participants with both high METS-IR and high BMR. Multivariate models were adjusted for age, gender, smoking status, drinking status, education level, marital status, residence, SBP, DBP, TC, HDL-C, LDL-C, hs-CRP, chronic diseases (hypertension, dyslipidemia, depression, and cancer), and medication use (antihypertensive, antidiabetic, antidyslipidemic, and cardiovascular medications), with the exception of the stratification variable


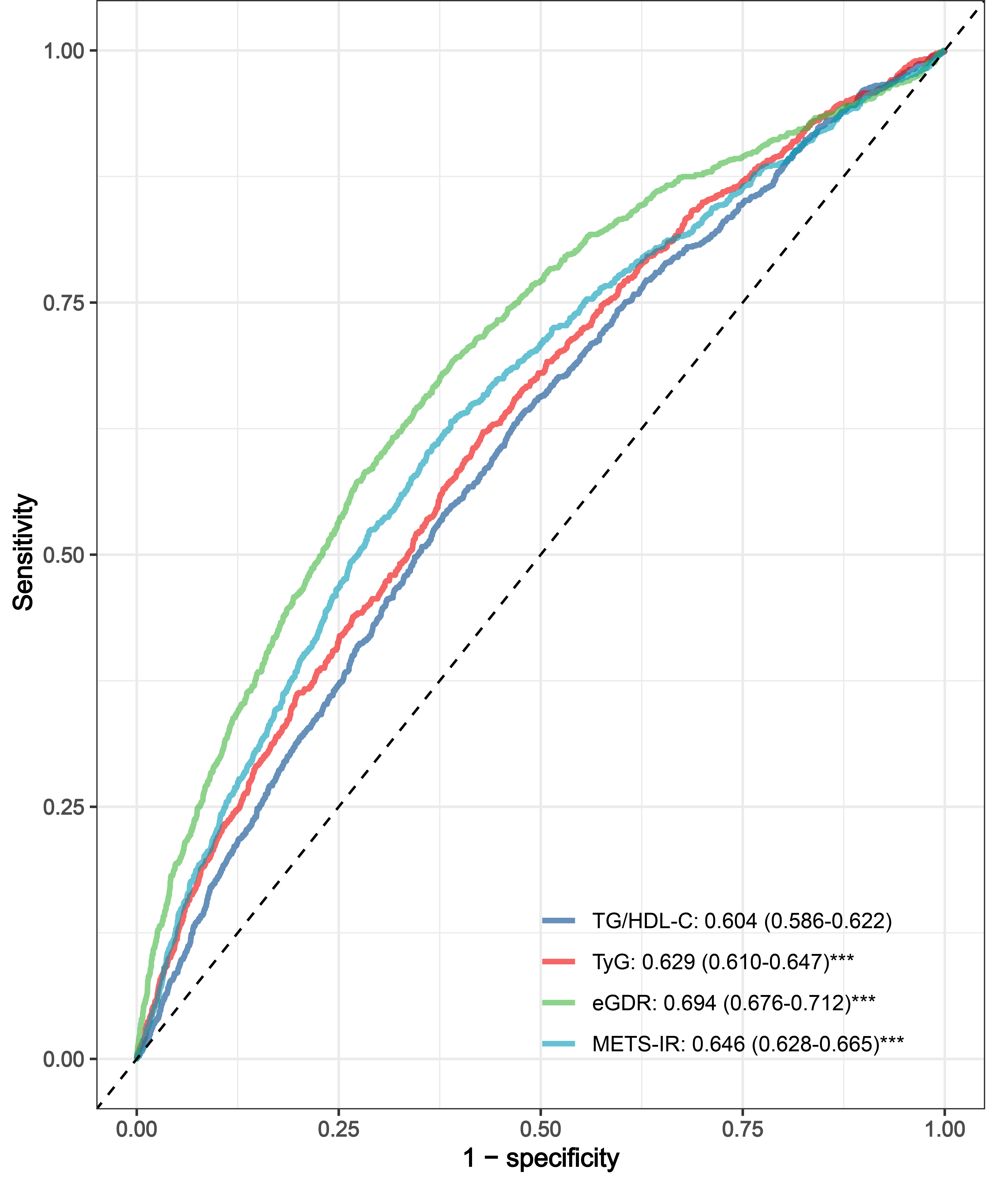


**Fig. S6** ROC curves for predicting cardiometabolic multimorbidity (CMM), stratified by insulin resistance (IR) assessment method. This figure compares the predictive performance of four IR surrogates: the TyG index, eGDR, METS-IR, and TG/HDL-C ratio. *** indicates significantly better predictive performance compared to the TG/HDL-C ratio by DeLong's test (P < 0.001)





**Fig. S7** Dose-response relationships of BMR with CMM risk. Spline analyses were adjusted for age, gender, smoking status, drinking status, education level, marital status, residence, SBP, DBP, TC, HDL-C, LDL-C, hs-CRP, chronic diseases (hypertension, dyslipidemia, depression, and cancer), and medication use (antihypertensive, antidiabetic, antidyslipidemic, and cardiovascular medications). *BMR* basal metabolic rate, *HR* hazard ratio, *CI* confidence interval


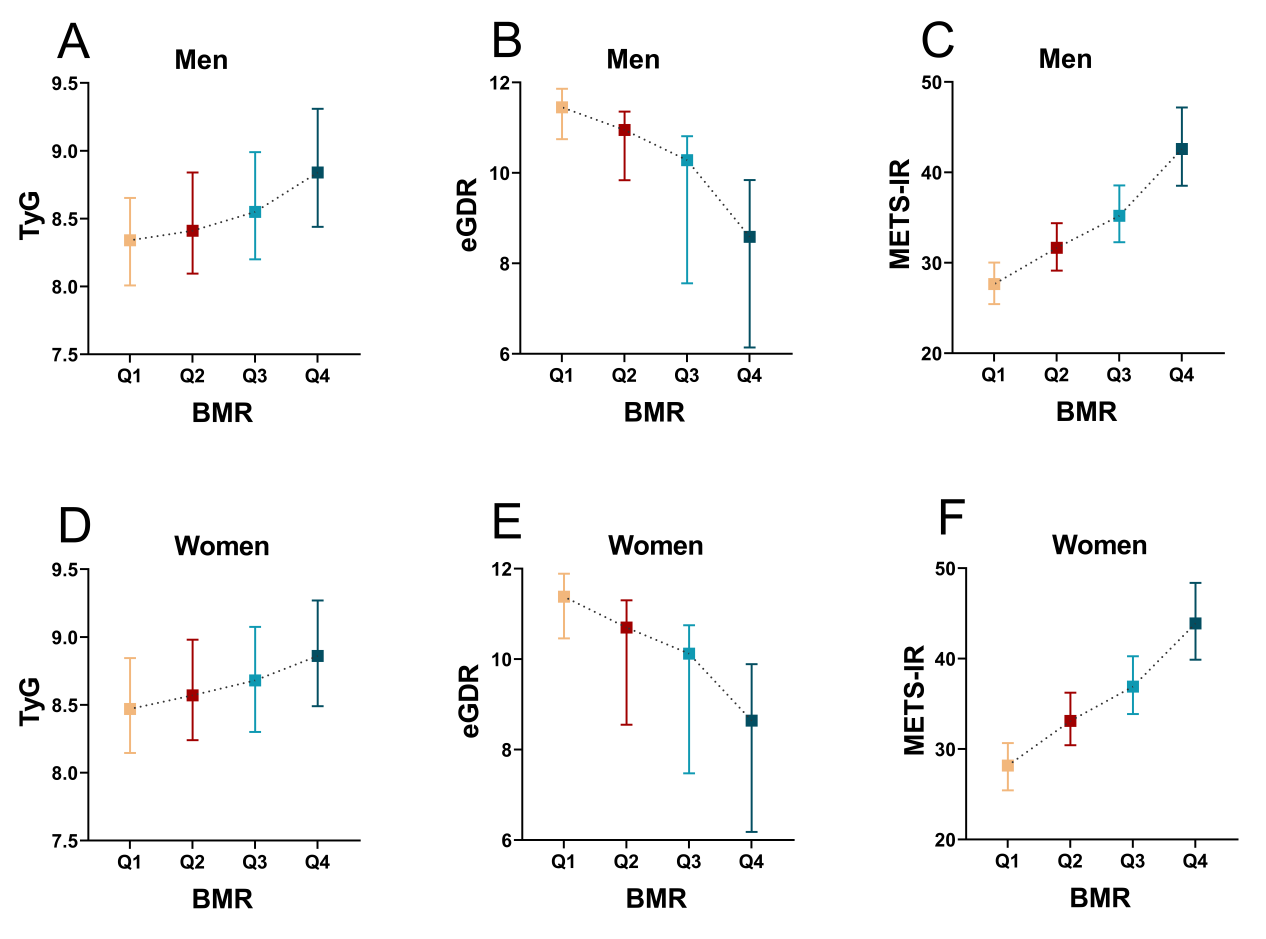


**Fig. S8** Levels of surrogate indices for IR stratified by BMR quartiles (A-F). *IR* insulin resistance, *BMR* basal metabolic rate, *TyG* triglyceride-glucose, *eGDR* estimate glucose disposal rate, *METS-IR* metabolic score for insulin resistance


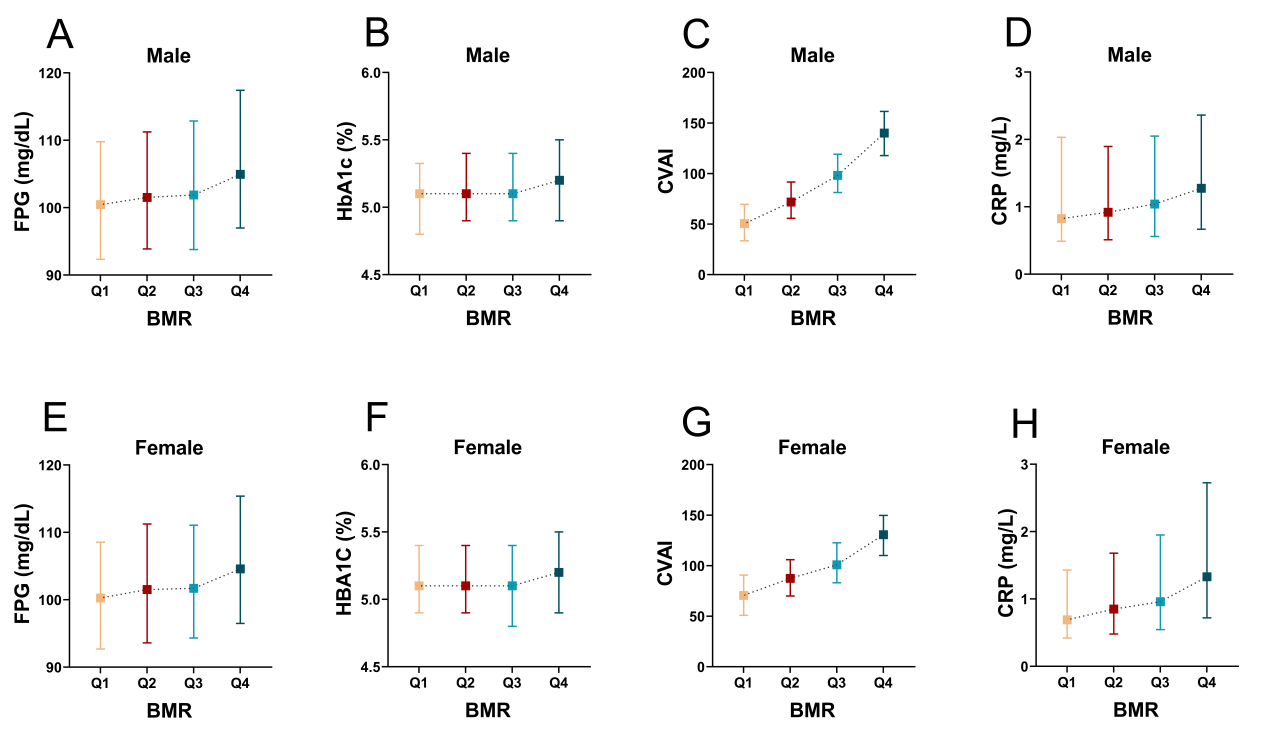


**Fig. S9** Levels of FPG, HbA1c, CVAI, and CPR stratified by BMR quartiles (A-H). *FPG* fasting plasma glucose, *CVAI* Chinese Visceral Adiposity Index, *CRP* C-reactive protein, *BMR* basal metabolic rate


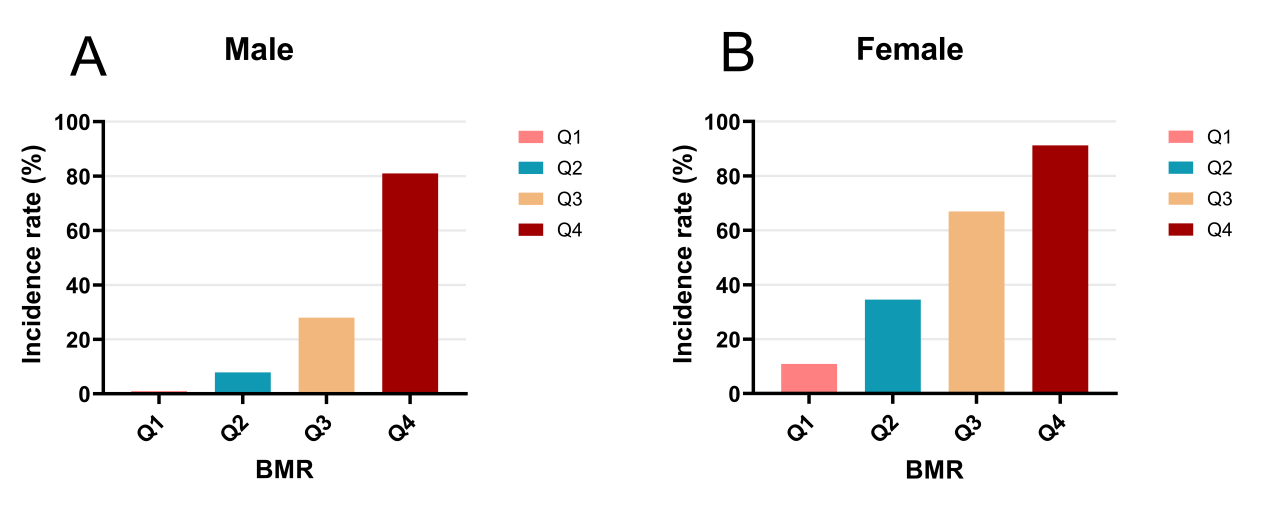


**Fig. S10** The incidence of abdominal obesity (AO) stratified by BMR quartiles (A-B). *BMR* basal metabolic rate. The waist circumference thresholds for diagnosing AO in male and female were set at ≥ 90 cm and ≥ 85 cm, respectively
